# Repeated evolution and the impact of evolutionary history on adaptation

**DOI:** 10.1186/s12862-015-0424-z

**Published:** 2015-07-10

**Authors:** Terry J. Ord, Thomas C. Summers

**Affiliations:** Evolution and Ecology Research Centre, and School of Biological, Earth and Environmental Sciences, University of New South Wales, 2052 Kensington, NSW Australia

**Keywords:** Parallel evolution, Convergent evolution, Phylogenetic distance, Taxonomic distance, Homoplasy, Many-to-one mapping

## Abstract

**Background:**

Whether natural selection can erase the imprint of past evolutionary history from phenotypes has been a topic of much debate. A key source of evidence that present-day selection can override historically contingent effects comes from the repeated evolution of similar adaptations in different taxa. Yet classic examples of repeated evolution are often among closely related taxa, suggesting the likelihood that similar adaptations evolve is contingent on the length of time separating taxa. To resolve this, we performed a meta-analysis of published reports of repeated evolution.

**Results:**

Overall, repeated evolution was far more likely to be documented among closely related than distantly related taxa. However, not all forms of adaptation seemed to exhibit the same pattern. The evolution of similar behavior and physiology seemed frequent in distantly related and closely related taxa, while the repeated evolution of morphology was heavily skewed towards closely related taxa. Functionally redundant characteristics—alternative phenotypes that achieve the same functional outcome—also appeared less contingent.

**Conclusions:**

If the literature provides a reasonable reflection of the incidence of repeated evolution in nature, our findings suggest that natural selection can overcome contingent effects to an extent, but it depends heavily on the aspect of the phenotype targeted by selection.

**Electronic supplementary material:**

The online version of this article (doi:10.1186/s12862-015-0424-z) contains supplementary material, which is available to authorized users.

## Background

The independent evolution of the same phenotype in different taxa implies to many observers that some underlying selection pressure has been shared among taxa to produce the convergent phenotype. While similarities in phenotype can arise independently in taxa by chance [1], there are many cases in which natural selection is responsible for repeated evolution [2–5]. Spectacular examples include the replicated morphologies that reflect similarities in habitat use among Caribbean *Anolis* lizards [6, 7] or three-spined stickleback fish [8]. Such examples are striking because they appear to override the idiosyncrasies inherent in the process of evolutionary differentiation [9]. For example, Gould [10] argued that evolutionary outcomes are contingent on a complex sequence of unique historical events that invariably leave an imprint on the phenotypes of descendent taxa. The vagaries of evolution will therefore lead phylogenetically divergent taxa to respond in different ways to similar selection pressures. There is extensive empirical and theoretic work to support this view [9, 11–15]. Yet cases of repeated evolution seem to refute it by presenting clear evidence that natural selection can override the contingent nature of evolution. This in turn suggests that the phenotypes of taxa are shaped by present-day ecology more so than past evolutionary events.

The seemingly conflicting views of historical contingency and ecological determinism have been the subject of much debate (reviewed by [4] and [16]). Supporters of Gould’s view might argue that *Anolis* lizards and stickleback fish—and most other examples of adaptive convergence for that matter—are cases where the same adaptation has evolved independently among *closely related taxa*. Members of the same genus or species tend to occupy similar environments [17] and tend to share key aspects of their genome or developmental pathways that can predispose them to follow similar evolutionary trajectories (e.g., [18, 19]). It is not surprising, then, that closely related taxa are often exposed to similar selection pressures or that they subsequently respond to those selection pressures in a similar manner.

The key point of contention then is time. Gould’s argument rests on the assumption that the signature of contingency will become greater the longer taxa evolve independently of one another. This predicts the likelihood of similar adaptations evolving independently in different taxa will decrease with the phylogenetic separation of those taxa. However, others might argue that the number of possible adaptations that might evolve in response to a given selection pressure is finite, and this will tend to “stack the deck” in favour of taxa evolving similar adaptations irrespective of the length of time those taxa have evolved independently of each other [20]. Consider the textbook examples of flight in birds and bats through the repeated evolution of the wing [21], or vision in vertebrates and cephalopods through the repeated evolution of a lensed eye [22]. In both instances, there are probably a limited number of adaptive options: some sort of wing or some sort of eye. There may also be developmental constraints that limit the pool of potential outcomes that can be expressed and bias organisms to converge on similar phenotypes [3, 5]. Whether adaptation is or is not contingent therefore has important implications for our understanding of the adaptive process and the predictability of evolution more generally.

There have been at least two investigations of the likelihood of repeated adaptive evolution as a function of evolutionary time. Conte et al. [23] found a general decrease in the occurrence of repeated adaptive evolution caused by the same genetic processes (the outcome of “parallel” evolution; Table 1) as the amount of evolutionary time separating taxa increased (see also [18, 19, 24]). Vermeij [20] adopted a different approach and examined incidences of putatively unique adaptations and repeated evolution of the same adaptation (“convergent” evolution; Table 1) over the entire history of life. He found an apparent increase in the incidence of unique adaptations coupled with a reduction in repeated evolution with increasing geological time. However, he argued that this almost certainly reflected the increasing difficulty in distinguishing unique from repeated evolution over vast stretches of geological time (e.g., gaps in the fossil record will increasingly present instances of repeated evolution as unique or entirely absent the further back in time those adaptations evolved). He concluded that adaptive evolution was not contingent on past evolutionary events and that convergent evolution has probably been frequent throughout the history of life [20]. Although the perspectives taken by these two studies were different, the discrepancy in their conclusions might also reflect the type of repeated evolution examined: parallel evolution in the first instance ([23]; Table 1) and the more broadly defined convergent phenotypic evolution in the second instance [20]. Parallel evolution is arguably less likely among distantly related taxa because the probability of two taxa sharing the same genetic mechanism presumably decreases with the length of time separating those taxa ([23]; but see [2]). When adaptations arise through different genetic pathways, however, the probability of convergent adaptations evolving is likely less contingent on the length of time separating taxa [5].

**Table 1:** Glossary of terms

Term	Definition
Convergent evolution	The independent evolution of a similar phenotype. In the context of this study, we focus specifically on phenotypic characteristics that achieve a similar adaptive outcome (i.e., are examples of *adaptive* convergent evolution), but phenotypic convergence can also arise through non-adaptive processes (e.g., see [1] and [3]). Ideally convergent evolution (adaptive or otherwise) is distinct from parallel evolution in that phenotypes have been generated from different genetic processes. However, this distinction cannot be made for most cases of reported convergence because the genetics that underlie characteristics have yet to be investigated. Convergent adaptations should also be distinct from those that are functional redundant, but in some cases it can be difficult to determine whether phenotypic characteristics are in fact similar or different among taxa.
Functional redundancy	The evolution of different phenotypes that achieve a similar functional outcome in different taxa. Also referred to as many-to-one form to function mapping. This is distinct from “incomplete” convergence in that divergent phenotypes are believed to be functionally equivalent and potentially adaptive.
Historically contingent	All organisms share a common ancestor at some point, but intervening factors such as past selection pressures, genetic drift, random mutation (and mutation order) and other stochastic factors (extrinsic chance events) direct the evolution of lineages along increasingly divergent trajectories as time progresses.
Parallel evolution	The independent evolution of similar genetic processes that produce a similar phenotype. As with convergent evolution, our survey focussed on characteristics that were believed to achieve a similar adaptive outcome (i.e., are examples of *adaptive* parallel evolution), but parallel evolution might also arise from non-adaptive processes. This is distinct from the classical definition still used by many researchers of the independent evolution of similar adaptations among taxa that share a close common ancestor (sensu [48]).
Repeated evolution	The independent evolution of a similar functional outcome in different taxa, either through the evolution of similar phenotypes (parallel and convergent evolution) or different phenotypes that achieve the same functional outcome (functional redundancy).

Our goal was to examine published examples of the various types of repeated evolution and whether the likelihood of similar adaptations arising through these was contingent on shared evolutionary history. To this end, we conducted a broad survey of reports of repeated evolution among animal taxa published over the last 10 years [NB: this assumed, like past studies (e.g., [18–20, 23]), that reports in the literature provide a reasonable reflection of the incidence of the repeated evolution of adaptations in nature; see discussion for an alternative interpretation of research bias and its implications]. If contingency in the adaptive process is strong, then the likelihood of repeated evolution will decrease with the increasing phylogenetic distance of taxa. Alternatively, if adaptation is free to vary independently of past evolutionary events, then the probability of repeated adaptive evolution when exposed to similar selection pressures should be just as likely among distantly related taxa as more closely related taxa. We tested these predictions in three types of repeated evolution (Table 1): (i) ‘parallel’ evolution in which adaptation is generated by the same genetic mechanism; (ii) ‘convergent’ evolution in which adaptation is likely the product of different genetic mechanisms; and (iii) ‘functionally redundant’ evolution in which different phenotypic forms serve the same functional outcome (also known as “many-to-one” mapping of form to function; see [25]). We predicted that reported examples of parallel and convergent evolution of adaptations would be less likely to occur among distantly related taxa, whereas adaptations that were functionally redundant would be equally likely or even more likely to evolve among distantly related taxa compared to closely related taxa. Finally, given that different aspects of an animal’s phenotype are potentially more or less “evolvable”, the extent to which the evolution of different aspects of the phenotype are historically contingent might also vary. We therefore examined the incidence of repeated adaptive evolution separately for the most commonly reported aspects of the phenotype found to evolve repeatedly (morphology, behavior and physiology).

## Results

### Type of animals and characteristics found to exhibit repeated adaptive evolution, and associated selection pressures

**Fig. 1:**
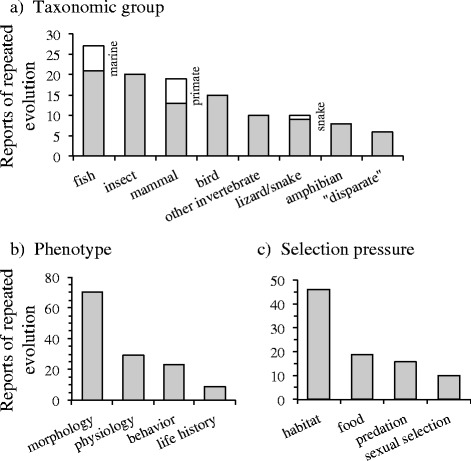
Published reports of repeated adaptive evolution. Examples by (**a**) taxonomic group, (**b**) aspect of the phenotype exhibiting convergence, and (**c**) selection pressure believed to have produced convergence. “Disparate” refers to repeated evolution among taxa across taxonomic groups (e.g., convergence between a bird and a lizard)

Fish were by far the most common taxa reported to exhibit adaptations arising from repeated evolution (23 % of examples), followed closely by insects and mammals (17 % in both cases; Fig. 1a). The vast majority of examples of repeated adaptive evolution were morphological (53 %), with instances of repeated adaptation less frequently reported for behavior (22 %) or physiology (18 %), and rarely for life history (7 %; Fig. 1b).

**Fig. 2:**
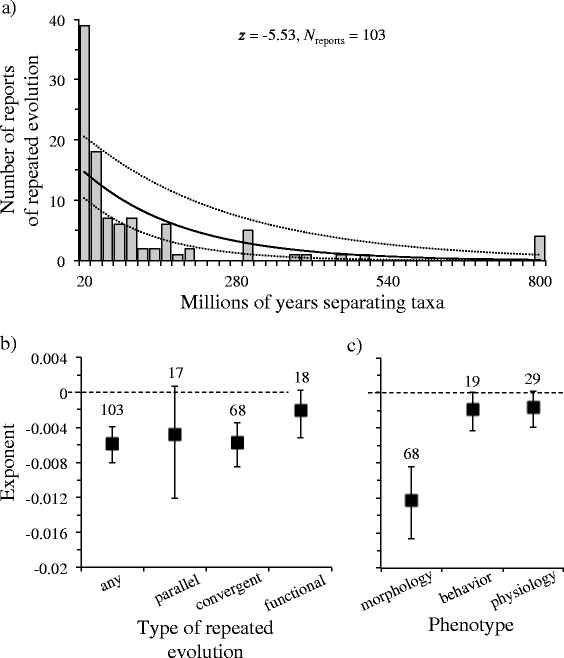
Incidence of repeated adaptive evolution. Reports of repeated evolution among taxa as a function of phylogenetic separation (**a**). Reports were also categorised by the type of repeated evolution involved (**b**) and phenotypic characteristic studied (**c**). Error bars associated with estimated exponents are 95 % confidence intervals. Numbers above error bars correspond to the number of reports found

Similarity in habitat type among taxa was clearly the predominant factor associated with the repeated evolution of similar adaptations in most reports (48 %; Fig. 2c). Exploiting similar types of food resources or dealing with similar types of predators were less frequently reported but still reasonably common selection pressures believed to have prompted the evolution of similar adaptations among taxa (21 % and 18 %, respectively). In contrast, sexual selection was rarely invoked to explain repeated evolution (<10 %).

### Likelihood of repeated adaptive evolution as a function of phylogenetic distance

Overall, the number of reports of repeated adaptive evolution dropped progressively with the increasing phylogenetic separation of taxa. This was the case irrespective of whether absolute time (Fig. 2) or taxonomic distance (Additional file 1: Figure S1) was used to gauge the separation of taxa, or whether the analysis was restricted to taxa with roughly similar generation times (Additional file 2: Figure S2). Nevertheless, there were important differences depending on the type of repeated evolution and phenotypic characteristic studied.

Examples of adaptations resulting from ‘parallel’ evolution tended to drop off with increasing phylogenetic distance, but the trend was weak (confidence intervals associated with the computed exponent overlapped zero; Fig. 2b and Additional file 1: Figure S1b). More pronounced was the reduction in the number of reports of ‘convergent’ evolution as taxa became increasingly separated from one another (represented by a negative exponent that was statistically different from zero; Fig. 2b, Additional file 1: Figure S1b and Additional file 2: Figure S2b). Adaptations described as ‘functionally redundant’ appeared least contingent on evolutionary history (Fig. 2b and Additional file 1: Figure S1b), with reports being frequent among both closely related and distantly related taxa.

Of the three most commonly studied phenotypic characteristics (morphology, behavior, and physiology), adaptations in morphology seemed heavily contingent on the length of evolutionary time separating taxa (and this result was robust to reductions in sample size in a reanalysis of 100 random subsets of the data; results not shown), whereas examples of repeated adaptive evolution in behavioral and physiological characteristics were less contingent (Fig. 2b, Additional file 1: Figure S1b and Additional file 2: Figure S2b; examples of the repeated evolution of life history were too few to allow a similar assessment).

**Fig. 3:**
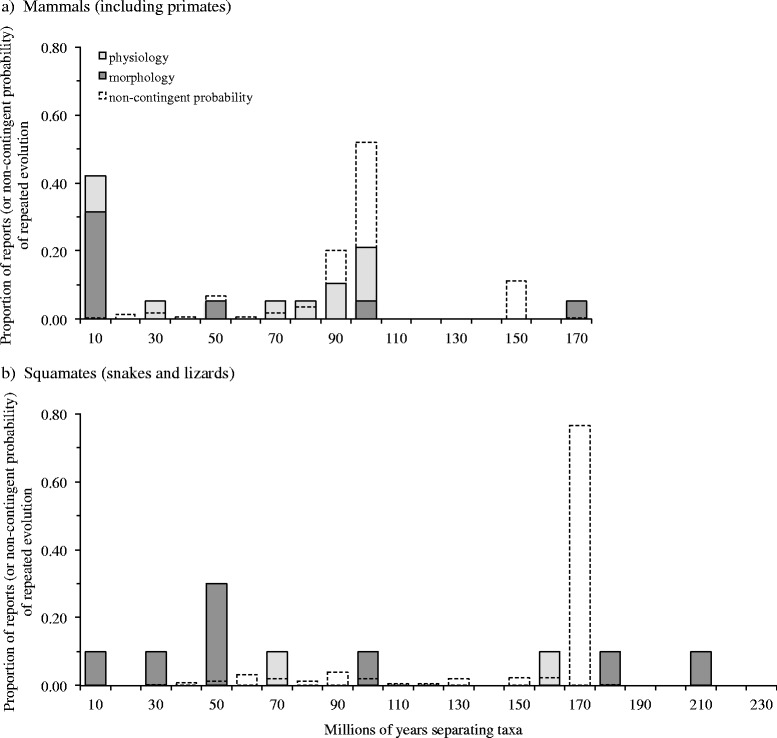
Proportion of reports of repeated adaptive evolution versus null distributions in two key taxonomic groups. Morphological and physiological repeat adaptations among mammalian (**a**) and squamate taxa (**b**) as a function of phylogenetic separation. Shown as dashed bars are the proportion of species pairs at different phylogenetic separations across the entire super-tree for each group. These provide a general estimate on the proportion of species that have the potential to exhibit repeated evolution, if adaptive outcomes were not contingent and taxa were exposed to similar selection pressures

Finally, our examination of the expected distribution of repeated evolution if adaptation was not contingent showed that incidences of repeated evolution should tend to be clustered among more distantly related taxa, simply because there are more distantly related taxa than closely related taxa to share potential adaptations (Fig. 3). That is, the incidence of repeated evolution should, in general, increase with the phylogenetic separation of taxa if not contingent on past evolutionary events. Given that the computed exponents in our meta-analysis were either negative or close to zero (i.e., none were positive; Fig. 2b, Additional file 1: Figure S1b and Additional file 2: Figure S2b), all types of repeated adaptive evolution (parallel, contingent, and functionally redundant) and all aspects of the phenotype reported to exhibit repeated adaptive evolution (morphology, behavior and physiology) seemed to be historically contingent to a lesser or greater degree. However, the interpretation of exponents that were not significantly different from zero may not be straightforward. The distribution of non-contingent repeated evolution will tend to differ depending on whether all or part of a phylogeny is considered. For example, the null distribution of non-contingent repeated evolution among taxa within squamate clades separated by less than 160 MY appears evenly distributed among closely related and distantly related taxa (Fig. 3b), which would equate to an exponent estimate of zero. However, consideration of the entire squamate phylogeny shows a prominent skew in the probability of repeated evolution towards taxa separated by more than 160 MY. Similar, disparate patterns in the distribution of non-contingent repeated evolution are also evident in clades of mammals separated by less or more than 80 MY (Fig. 3a).

### Factors associated with historically contingent adaptation

**Fig. 4:**
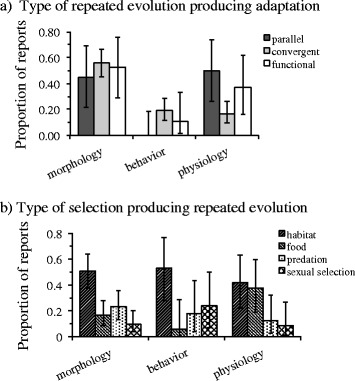
The degree to which different aspects of the phenotype exhibit similar adaptations through (**a**) parallel, convergent, or functional redundant evolution or (**b**) similarities in selection pressure. Error bars are 95 % confidence intervals computed from equations found in [47]

Given the clear skew in the repeated evolution of morphological adaptations towards closely related taxa compared to the seemingly less contingent evolution of behavior and physiology, we examined whether there were any obvious differences among these phenotypic characteristics and the type of repeated evolution or selection pressure involved in generating adaptation. There were no noticeable differences in either the type of repeated evolution or selection pressure reported between morphological, behavioral or physiological adaptations. In general, all aspects of the phenotype were produced by parallel, convergent and functional redundant evolution in roughly equal proportions (Fig. 4a). There was also little difference in the type of selection pressure reported to have generated repeated adaptation among phenotypic characteristics, (e.g., morphology, behavior and physiology were roughly equal targets of selection resulting from similarities in the type of habitat; Fig. 4b).

## Discussion

If the literature provides a reasonable reflection of the incidence of repeated adaptive evolution in nature, our results imply that closely related taxa tend to be predisposed to adapt in similar ways, and that Gould [10] was generally right: evolution does not tend to repeat itself over large macroevolutionary time scales. Nevertheless, there was important variation underlying this general trend that provides further insight on how important contingency is in the outcome of adaptive evolution.

First, the inverse relationship between the likelihood of repeated adaptive evolution and the phylogenetic separation of taxa was not linear, regardless of whether time or taxonomic level was used to categorise the degree of separation of taxa. This implies the influence of evolutionary history on the trajectory of evolution has a disproportionately greater impact on evolutionary outcomes over the longer term than the short term (see also [20] and [24]). This was unexpected, but the pattern may be broadly consistent with a Brownian motion mode of evolution. Under this model, phenotypes are expected to change gradually, with the accumulation of phenotypic change occurring largely constant over evolutionary time (or at least not vary systematically over different time scales; reviewed by [26]). While this means the phenotypic distance of two lineages originating from a common ancestor will tend to be proportional to the evolutionary time separating the two taxa, the magnitude of phenotypic separation between those taxa will scale as a square-root function of time (see [27, 28]). If one of those taxa happens to be near an adaptive peak, the distance of the other taxon from that same peak will be, on average, the square-root of the length of time separating the two taxa from a common ancestor. That is, distantly related taxa will tend to be disproportionately further away from a common adaptive peak than closely related taxa. Similar patterns are also expected under alternative modes of evolution such as Ornstein-Uhlenbeck, although the disproportionate influence of time on phenotypic distance will likely be even greater [27].

Alternatively, most adaptations (or at least those reported to have evolved repeatedly in different taxa) might have a complex genetic architecture associated with their origin or are subject to developmental constraints that result in a non-linear change in the probability of convergence as a function of time. Natural selection might produce similar adaptations in different taxa separated by less than 40 MY (Fig. 2a) or among members of the same species or genus (Additional file 1: Figure S1a), but over longer periods of separation, any adaptive resemblance among taxa becomes unlikely. For example, there are potentially important differences in the genetic mechanisms that cause phenotypic variation among intraspecific and interspecific taxa (especially in morphology [18, 19]; see also [24]). At this stage, however, how these differences or some other aspect of the genetics or development of adaptation might influence repeated evolution over different phylogenetic scales is unclear. Furthermore, although nearly half of the reported cases of parallel adaptive evolution included in our meta-analysis occurred among taxa separated by less than 20 MY (within the same species or genus), there were still cases reported among taxa separated by more than 80 MY (species from separate families; e.g., parallel evolution of high-frequency hearing in echolocating bats and cetaceans [29]). This at least shows that the evolution of similar adaptations is possible through similar genetic changes among distantly related taxa (see also [2]).

Second, the extent to which adaptations were contingent on evolutionary history appeared to depend on the specific aspect of the animal’s phenotype under selection. The evolution of similar morphologies appeared to be more contingent than either behavior or physiology. For example, the incidence of repeated adaptive morphological evolution among mammals sharing a common ancestor of up to 10 MYA was almost 80 times more likely than would be expected if repeated evolution was not contingent (Fig. 3a). In contrast, the distribution of reports of repeated adaptive evolution in physiology were more similar to that expected for the outcome of adaptations unaffected by evolutionary history (Fig. 3a). Either researchers of morphology have approached the study of repeated evolution in a fundamentally different manner to behavioral ecologists and physiologists (e.g., morphologists are more strict on how similar adaptations must be to be considered an example of repeated evolution, or tend to focus on more closely related taxa for evidence of convergence than distantly related taxa), or the process of morphological adaptation differs in important ways to that of behavior and physiology. For example, behavior and physiology are often viewed as being more evolutionary labile than morphology (e.g., [30]). Plasticity might also increase the ability of taxa to adapt their behavior or physiology to environmental changes in ways that increase the probability of more distantly related taxa achieving similar adaptive phenotypes. If we assume that researchers are studying repeated adaptive evolution in similar ways (and we have no reason to believe that they are not), then our findings suggest that behavior and physiology exhibit a higher degree of adaptability than morphology.

Finally, the result that functionally redundant adaptations exhibit less contingency than other adaptations suggests the adaptive response of distantly related taxa exposed to similar selection pressures might often be to evolve innovations in phenotype that achieve the same adaptive result (e.g., the evolution of different jaw morphologies in fishes that result in the same increase in feeding performance for a given food type; [25, 31]). Here, the notion that repeated evolution limits the evolution of phenotypic diversity is relaxed [31]. Intuitively, it might seem obvious that distantly related taxa would be more prone to evolve functionally redundancy simply on the basis that the criteria for classifying this form of repeated evolution requires only a demonstration that a characteristic is functionally equivalent and not also similar in form between taxa. However, taxa exhibiting functional redundant characteristics must still experience similar selection pressures in the same way as taxa that are found to have converged in phenotype as well. The likelihood that such similarities in selection occur among taxa presumably decreases with increasing phylogenetic separation, which will tend to coincide with taxa diverging in ecology and biogeography. That is, it is simplistic to assume that functionally redundant adaptations should not exhibit contingency in their evolution, and our survey also confirmed that it frequently evolves among closely related taxa (e.g., among populations) that do share much of their genome and developmental pathways (Additional file 3: Table S1; e.g., [32]).

It is important to note as well that adaptations classified as functionally redundant should not be discretely classified from adaptations more “typical” of classical convergence (e.g., the same morphological changes for the same adaptive function). Rather the phenomena of functional redundant, convergent, and parallel evolution is more appropriately viewed as a hierarchy in which the evolution of similar functions (adaptive or otherwise) are achieved through increasingly specific ways (function only → function through similar phenotypes → function through similar phenotypes generated from similar genetic changes; see [3]).

## Conclusions

The same adaptive solution evolving independently in different taxa provides a powerful illustration of natural selection in nature. Not surprisingly, then, documenting repeated evolution and confirming its adaptive origin has been the subject of much research effort, with over a hundred examples reported for animals alone over the last decade or so (and our survey was by no means exhaustive; see also [2–5, 20, 23]). Our meta-analysis of these examples showed the likelihood of repeated adaptive evolution appears to diminish as taxa become increasingly more phylogenetically divergent from each other, and dramatically so for some forms of adaptation (those that are morphological).

However, to fully understand the evolutionary contingency of the adaptive process, examples across all types of repeated evolution need to be considered (functionally redundant, convergent, and parallel). Although we found a large number of reports of adaptive convergence in the literature, the limited representation of functionally redundant adaptations (less than 20 % of papers reviewed and requiring a much broader search of the literature beyond the last decade), as well as the clear bias of morphological examples of convergence (over 60 %), is unlikely to reflect biological reality given that all aspects of the phenotype are subject to adaptive evolution. This indicates a broader perspective in the study of repeated adaptive evolution beyond morphology is clearly warranted.

It should also be considered that the nonlinear decrease in reports of repeated adaptive evolution with increasing phylogenetic separation might actually reflect that researchers are heavily biased towards studying adaptation among taxa belonging to the same species or genus. To some extent, this is to be expected given that most comparative biologists focus their investigations on particular taxonomic groups rather than a diverse range of taxa across the tree of life. Only the most striking cases of repeated evolution would therefore be recognised among highly disparate groups (e.g., the evolution of beaks in turtles and birds [33]; albinism in cave insects and fish [34]). Cases of repeated adaptive evolution in distantly related taxa might also have been a greater focus of classical studies of convergent evolution because it was easier to conclude independent evolution when taxa belonged to vastly different taxonomic groups (e.g., the convergent evolution of wings in birds and mammals – see Introduction). With the proliferation of molecular phylogenetic techniques and statistical advances in phylogenetic comparative methods over the last decade—a period of research that was the predominate focus of our meta-analysis—the study of repeated adaptive evolution may have shifted primarily to the study of closely related taxa. This seems unlikely given these same methodological advances have allowed phenotypic comparative analyses to be conducted at unprecedented phylogenetic scales (e.g., [7, 35–37]). It is nevertheless possible that many examples of repeated evolution in distantly related taxa remain undocumented. If this were the case, the impact of evolutionary history on adaptation documented in our analyses could be overestimated. More generally, such bias would represent a critical impediment to fully understanding the predictability of evolution and the process of adaptation at macroevolutionary time scales. Natural selection may in fact erase the signature of past evolutionary events from phenotypes more readily than our analyses imply, or genetic and developmental constraints may play a far greater role in adaptive evolution than currently appreciated [3, 5].

However, any publication bias in reported examples of repeated adaptive evolution would presumably affect all forms of repeated evolution and all types of phenotypic characteristics studied. That is, the same skewed pattern in the incidence of repeated adaptive evolution among closely related taxa that is so obvious for morphology (Fig. 2c and Additional file 1: Figure S1c) should be readily apparent in all of our data: i.e., parallel, convergent and functionally redundant examples, and morphological, behavioral and physiological characteristics should all exhibit the same general pattern. This was not the case and our results instead showed prominent—and predicted—differences in the incidence of repeated adaptive evolution across different forms of repeated evolution and different phenotypic characteristics. We believe, then, that our results are broadly reflective of the adaptive process in nature.

Some insight on potential research biases might be obtained by a future investigation that applies a quantitative estimate of adaptive similarity among taxa as a function of phylogenetic distance (e.g., [38]). This type of meta-analysis would not rely on count data and would potentially avoid any skew that might be generated by the types of organisms selected for study by comparative biologists. If enough examples could be obtained, a similar pattern in which the strength of convergence diminishes with phylogenetic distance would be particularly convincing support of Gould’s position on adaptive evolution. Given the increasing availability of data in public repositories, we anticipate that this type of meta-analysis should be achievable in the near future.

## Methods

### Literature survey

We searched the ISI Web of Science database from 2003 to 2012 using the topic search terms “converg* evolution”, “parallel evolution”, “homoplas*”, “functional* redundan*”, and “many-to-one” refined to the categories of evolutionary biology and ecology, and excluding plant sciences. Restricting our search to the last decade and by topic area was necessary because of the volume of articles uncovered (see below). Although our survey was not exhaustive (and it may have also missed examples of repeated adaptive evolution specific to aggressive mimicry or aposematic coloration; but see Additional file 3: Table S1), it should still provide a reasonable overview of the recent literature on repeated evolution. Searches were performed between April 24 and June 13 2013. Of the 2,602 articles found, we excluded review articles, conference abstracts, opinion pieces and book chapters. The titles and abstracts of the remaining articles were examined in detail and those that were found to be relevant animal examples of repeated evolution were downloaded through the University of New South Wales library (96 papers, see Additional file 3: Table S1; NB: papers for which electronic copies could not be obtained were not included in our analyses). Two papers that we were aware of and published in 2013 were also included (i.e., [39] and [40]).

We assessed each downloaded paper to confirm that the report was of a compelling case of repeated adaptive evolution in extant taxa. Specifically, that a functionally equivalent phenotypic characteristic evolved independently in different lineages and was likely to be the product of natural or sexual selection based on an empirical study reported in the article or the citation of a previous study in which the adaptive function of the characteristic had been reputedly assessed. We restricted our survey to extant taxa because of the problems of adequately identifying examples of repeated evolution in extinct animals (see [20] for discussion). Of those articles meeting our criteria, we then compiled information on the type of phenotypic characteristic involved, the taxonomy of the animals, the likely selection pressure driving the repeated evolution, and classified whether the adaptation was an example of parallel, convergent, or functional redundant evolution based on the definitions given in Table 1. For example, adaptations classified as examples of parallel evolution were those in which some aspect of the genetic pathway underlying the characteristic had been shown to be shared among taxa. It should be noted, however, that the vast majority of studies uncovered by our literature search did not examine the genetics of repeated evolution (87 of 96 articles). Given this, there were almost certainly cases classified as convergent that may have in fact originated through parallel evolution and have yet to be determined as such (see Table 1). To help bolster our coverage of cases in which the genetics of repeated adaptive evolution had been investigated, we included 14 additional cases identified by Conte et al. ([23]; 11 of which were confirmed cases of parallel evolution). This earlier study included nine other examples that were either already included in our data set (seven) or were specific to plants (two).

In the case of functionally redundant adaptations, many authors did not distinguish such adaptations from classical convergence. We therefore classified these examples based on whether the phenotypic characteristics reported to be functionally convergent were likely to be the same or different based on character descriptions presented in papers. In some cases, similar adaptations classified as convergent may in fact be more broadly functionally redundant. For example, Caribbean *Anolis* ecomorphs share key morphological characteristics such as particular limb lengths depending on the size of the perches used by a species belonging to an ecomorph category (reviewed by [41]). However, changes in limb length might have occurred in a variety of ways, such as increases in the femur or tibia, or both. Unless differences in the phenotypic characteristics were clearly described in the article, we classified examples as convergent, but point out—as with the case of distinctions between parallel and convergent—that these classifications may change as additional information becomes available with future research. Finally, we also contacted two experts familiar with the phenomenon of functional redundancy who provided us with additional examples that were not uncovered during our initial literature search (NB: four of these studies were published before 2003 and we included these in an effort to increase our sample size).

We used two estimates of the phylogenetic distance separating convergent taxa. First, we obtained an estimate in millions of years, either as reported directly in the paper or from a reference cited in the paper. Where this was not found, we used the mean estimate of time since divergence from TimeTree [42] based on a search of species or genera names (see Additional file 3: Table S1). Second, we used the taxonomic separation of reported taxa. For example, the taxonomic separation of Caribbean *Anolis* lizards convergent in morphology [43] was ‘genus’, whereas the maximum taxonomic separation of lizards convergent in morphology from the genera *Holbrookia*, *Sceloporus* and *Aspidoscelis* [44] was ‘order’. Although estimates of time since divergence increased with the taxonomic separation of taxa, the relationship was noisy and non-linear (see Additional file 4: Figure S3). There were also a handful of examples for which we were unable to obtain time estimates on separation, but were able to determine taxonomic separation that allowed these examples to be included in at least some of our analyses (those reported in Additional file 1: Figure S1). We therefore used both measures of phylogenetic separation in our analyses, but focussed primarily on time since divergence given it avoided the potential subjective biases of taxonomic classification.

### Meta-analysis

We counted the number of reported cases of repeated adaptive evolution in time bins of 20 MYA. Preliminary analyses showed this binning provided the best resolution of distribution patterns (NB: results were qualitatively similar using time bins of 5, 10, and 30 MYA). Counts of repeated adaptive evolution by taxonomic separation were made by converting taxonomic classifications into a score ranging from 1 (species) to 11 (kingdom; see Additional file 3: Table S1 and Additional file 4: Figure S3). Time bins or taxonomic categories in which no report of repeated evolution was found were treated as missing data rather than evidence for lack of repeated evolution among taxa at that phylogenetic separation. In some instances, several different papers reported repeated adaptive evolution in different characteristics among taxa from the same species group (e.g., *Anolis* lizards or stickleback fish). In these cases, the species group was used only once, either for the given type of characteristic being analysed (e.g., a behavioral characteristic in analyses of behavioral convergence – see below) or the earliest publication for that species group in analyses of broad trends (e.g., those in Fig. 2a). This ensured that highly studied groups did not skew our analyses and that the number of reports examined reflected convergence among different taxa, rather than the number of characteristics studied for the same group or the number of times the same characteristic has been studied for a species group.

To assess statistical trends in the distribution of reports of repeated adaptive evolution as a function of phylogenetic separation, we applied generalized linear models with a poisson error distribution (commonly known as a ‘count regression’) using R ver 3.0.2 (R Development Core Team). From these models, we compared the computed slope and effect size (*z* value) to evaluate the influence of phylogenetic distance on the probability of repeated evolution; however full model outputs are also provided in Additional file 5: Table S2. The diversity of taxa included in these analyses represented an equally diverse range of generation times. However, taxonomic groups representing short generation times (e.g., insect or fish) or long generation times (e.g., mammals) were well represented across all divergence times and taxonomic distances (fig. S1). That is, patterns of repeated adaptive evolution were unlikely to have been skewed by an over representation of certain groups with short or long generation times clustered at particular phylogenetic distances. Nevertheless, to confirm our findings were consistent, we conducted a separate set of analyses on instances of repeated evolution in fish, which were the largest taxonomic group represented in our data set (Fig. 1a) and were broadly similar in their generation times.

To further benchmark our findings, we also estimated the expected distribution of repeated adaptive evolution if its occurrence was unrelated to the phylogenetic separation of taxa (i.e., historically contingent effects on the outcome of adaptation were absent). Here, the likelihood of repeated evolution should be proportional to the number of potential species-pairs across the phylogeny. At the outset, we can make the general prediction that instances of repeated evolution should tend to be clustered among distantly related taxa rather than closely related taxa simply because there are more distantly related species pairs than closely related pairs on any phylogeny. Nevertheless, the specific distribution of potential species pairs will depend on the general properties of the phylogeny, in particular the age and frequency of rapidly radiating lineages within the tree (e.g., see [28]). Rather than use an artificially generated phylogeny, we chose two large time-calibrated phylogenies for mammals [45] and squamates (snakes and lizards; [36]). We reasoned that these would provide a more realistic and representative picture of probable patterns of repeated adaptive evolution on the tree of life than those obtained from a contrived phylogeny. We selected the phylogenies of mammals and squamates because these represented key taxonomic groups covered by our meta-analysis, included a large and diverse range of species (5,020 and 4,162 species, respectively), were time calibrated and species-level phylogenies (rather than genera or family level phylogenies), and could be readily downloaded from the supplementary information of each source.

We computed the length of time between all possible combinations of species pairs by extracting the variance-covariance matrices for each phylogeny using the R package ‘caper’ ver 0.5.2 [46]. The distribution of these distances were then plotted to provide an estimate on where instances of repeated evolution should be concentrated if the evolution of similar adaptations in different taxa were unrelated to the length of time separating taxa.

## Availability of supporting data

The data set supporting the results of this article is included within the article (and its additional files).

## Additional files

Additional file 1: Figure S1. Reports of repeated evolution among taxa as a function of taxonomic separation (**a**). Reports were also categorised by the type of repeated evolution (**b**) and phenotypic characteristic studied (**c**). Abbreviations are as follows: ‘sp’, species; ‘gen’, genus; ‘subfam’, subfamily; ‘fam’, family; ‘subord’, suborder; ‘ord’, order; ‘subcl’, subclass; ‘cl’, class; ‘subphyl’, subphylum; ‘phyl’, phylum; ‘subking’, subkingdom. See Fig. 2 for other details. Additional file 2: Figure S2. Reports of repeated evolution among fish taxa as a function of phylogenetic separation (**a**). Reports were also categorised by the type of repeated evolution (**b**) and phenotypic characteristic studied (**c**). See Fig. 2 for other details. Additional file 3: Table S1. Reported examples of adaptive convergence published over the last decade. Additional file 4: Figure S3. The relationship between time and taxonomic level separating convergent taxa. Also shown are individual plots for major taxonomic groups included in the meta-analysis. Additional file 5: Table S2. Full model outputs from analyses presented in figures.
