# Supplementary figures and images for: Repeated evolution and the impact of evolutionary history on adaptation
Source: BMC Evol Biol. 2015 Jul 10;15:137. doi: 10.1186/s12862-015-0424-z (PMC4497378; doi:10.1186/s12862-015-0424-z)

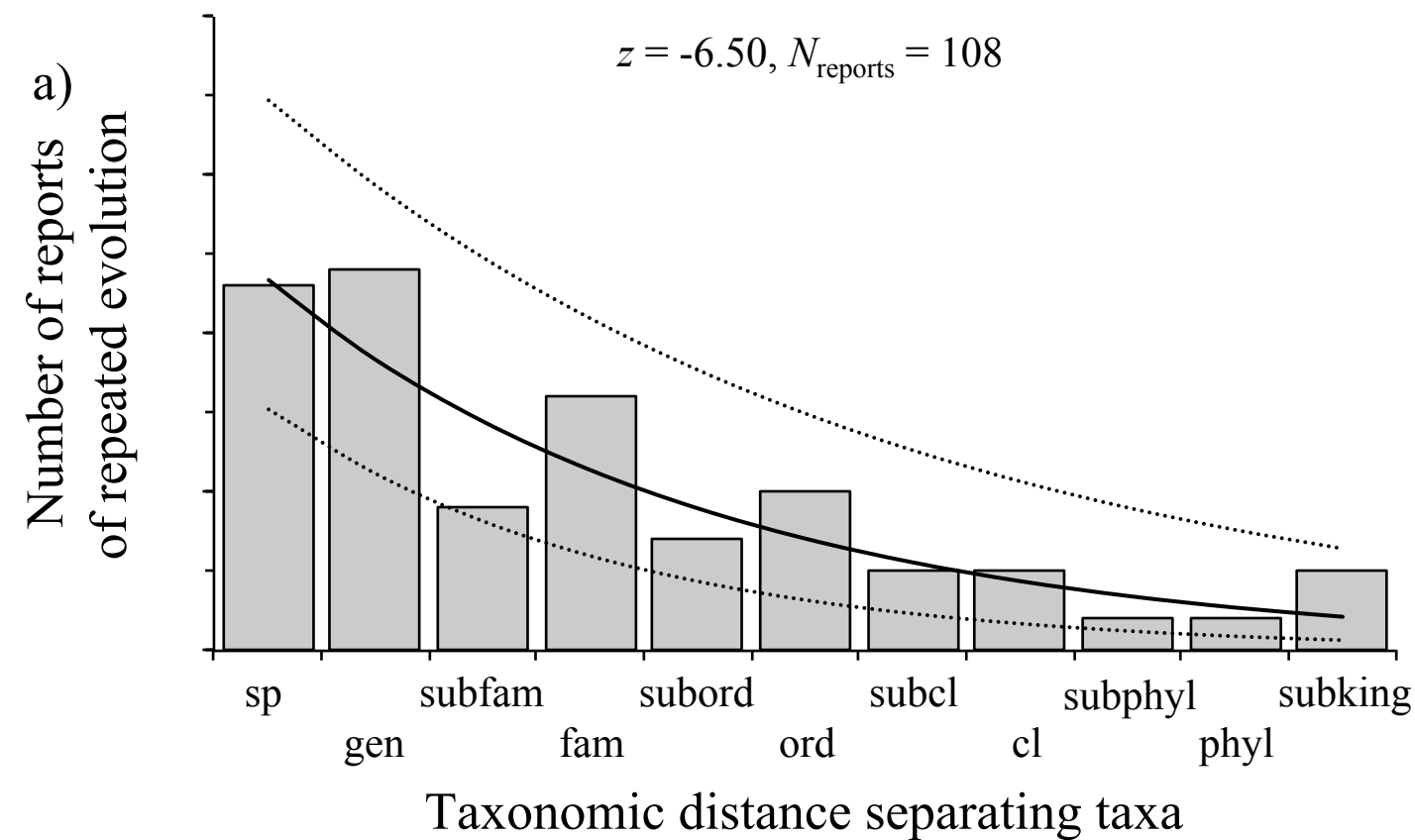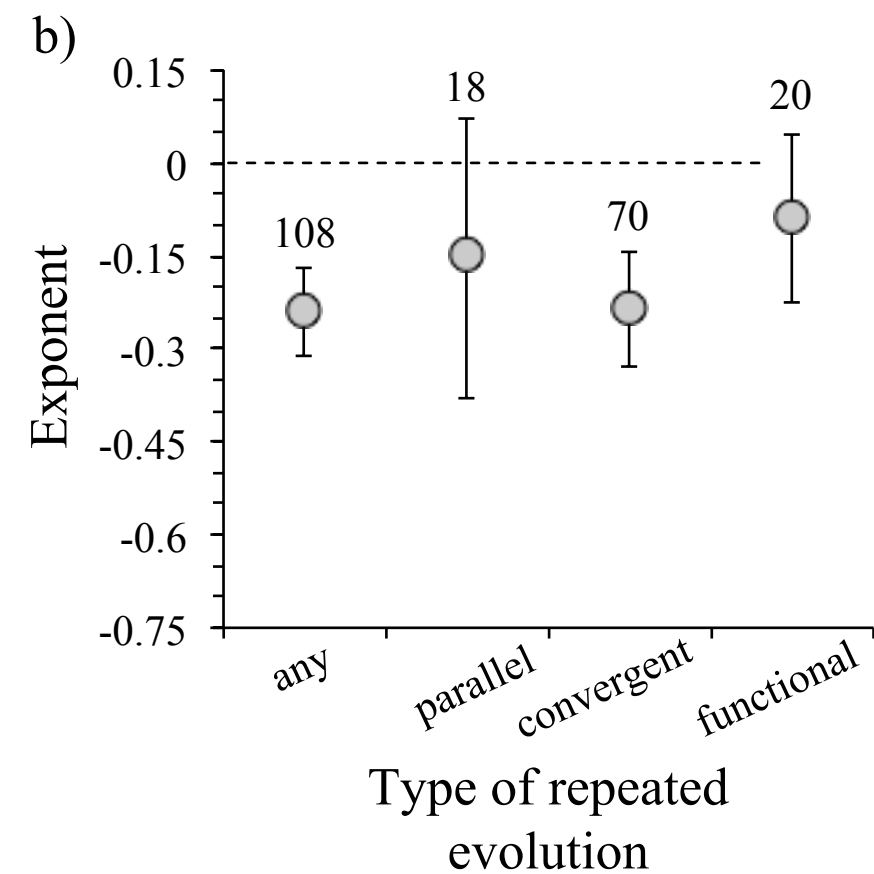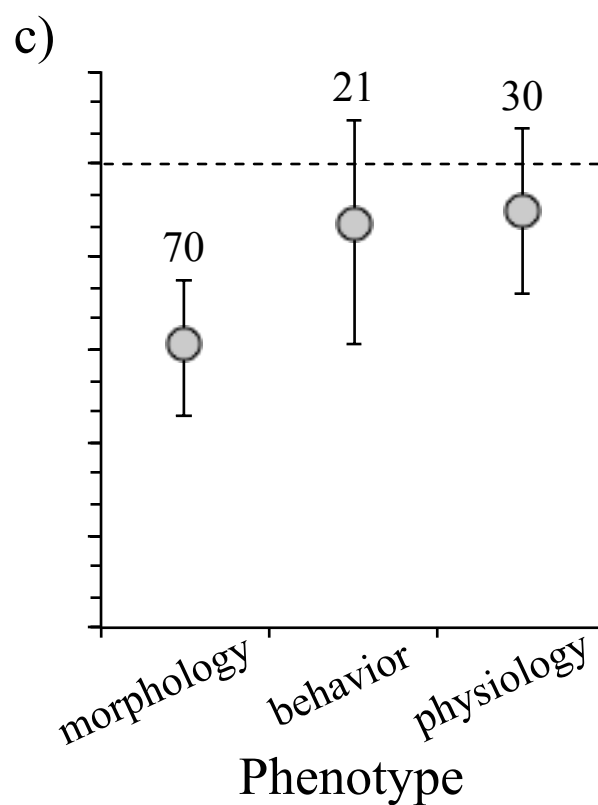

Supplement: Additional file 1: Figure S1. — Reports of repeated evolution among taxa as a function of taxonomic separation (a). Reports were also categorised by the type of repeated evolution (b) and phenotypic characteristic studied (c). Abbreviations are as follows: ‘sp’, species; ‘gen’, genus; ‘subfam’, subfamily; ‘fam’, family; ‘subord’, suborder; ‘ord’, order; ‘subcl’, subclass; ‘cl’, class; ‘subphyl’, subphylum; ‘phyl’, phylum; ‘subking’, subkingdom. See Fig. 2 for other details. [file 12862_2015_424_MOESM1_ESM.pdf]

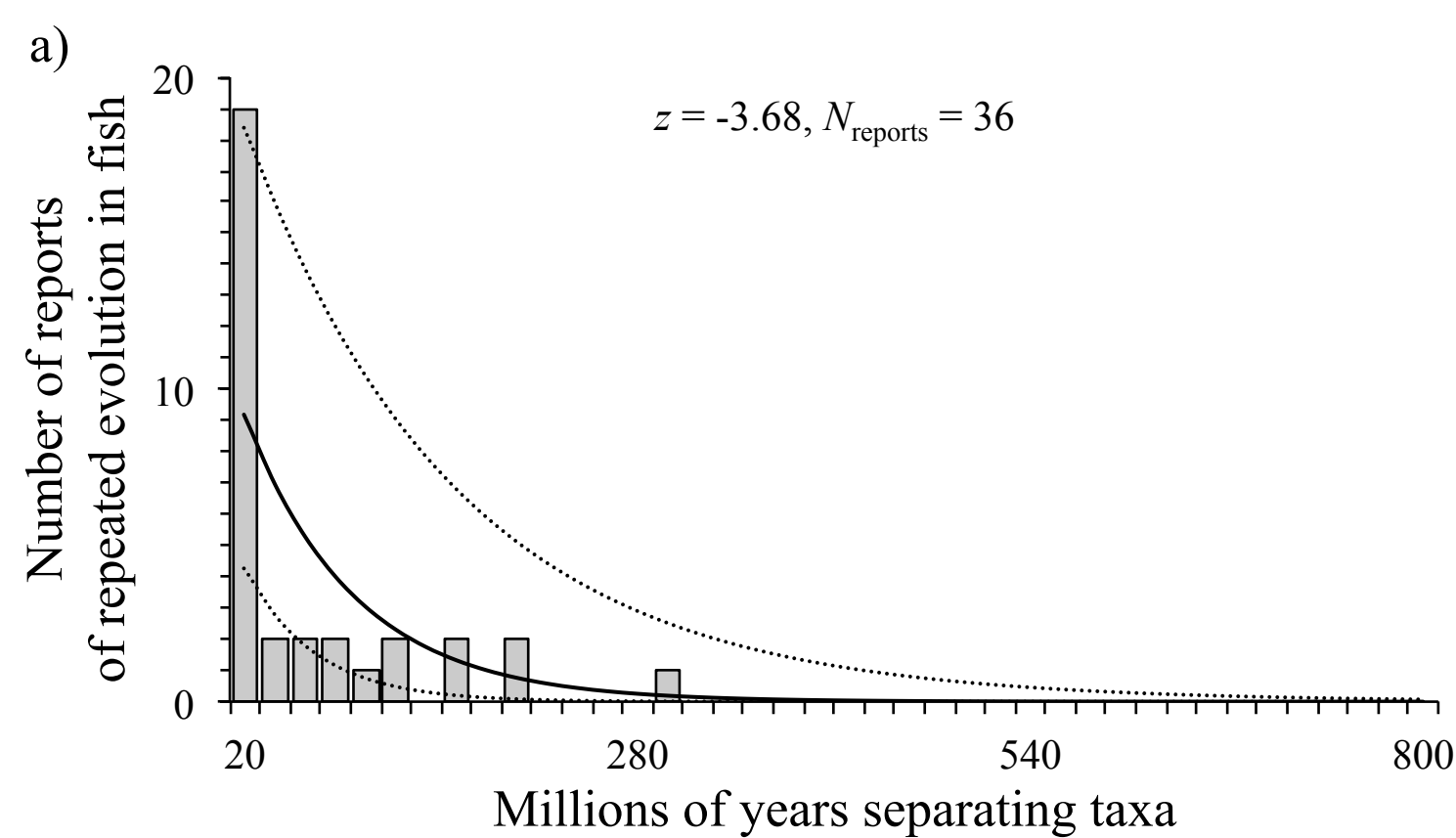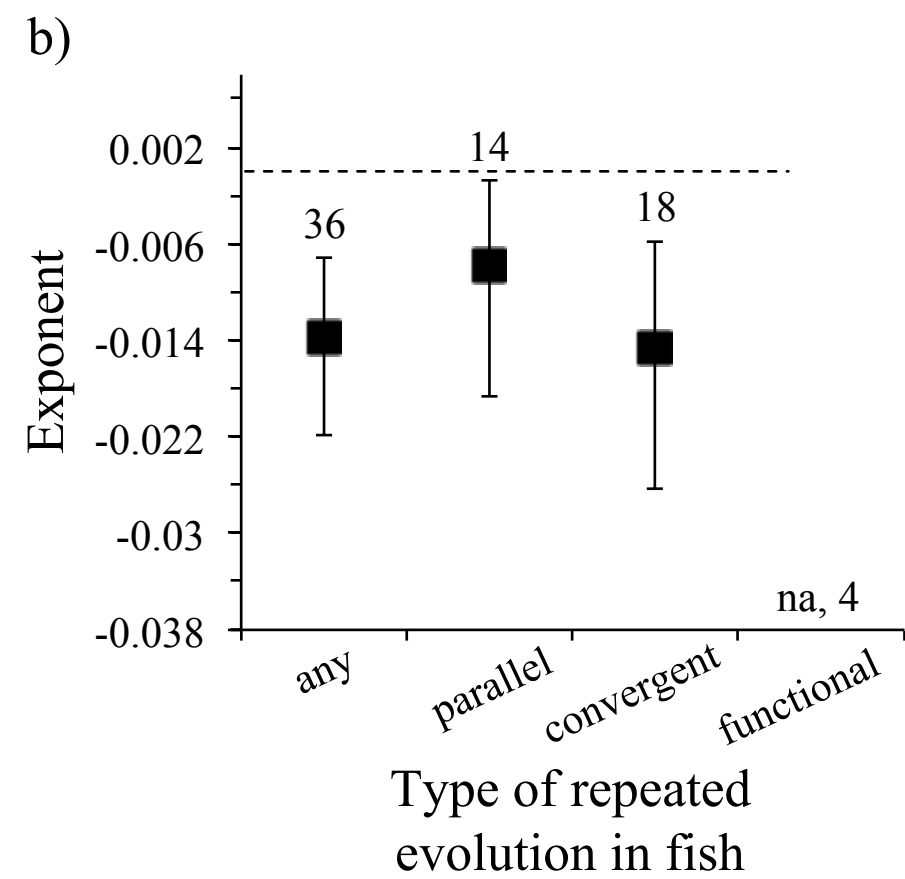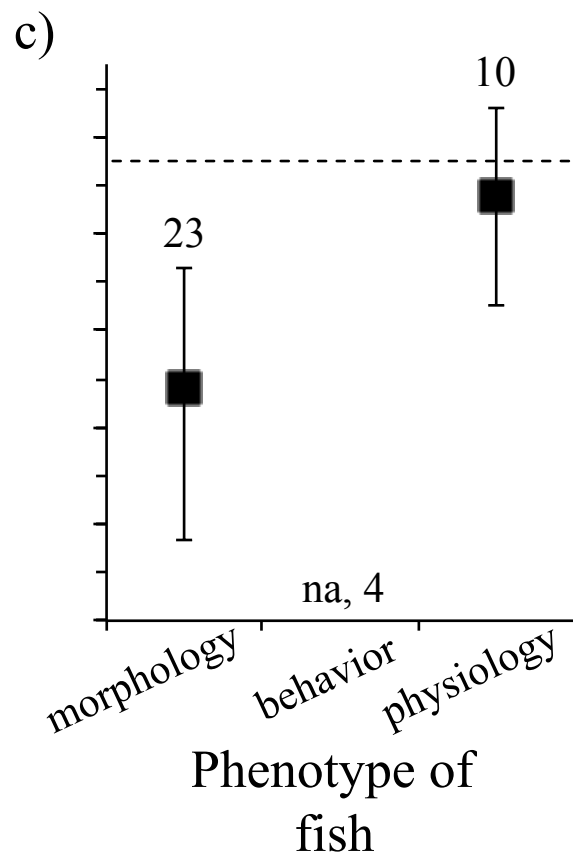

Supplement: Additional file 2: Figure S2. — Reports of repeated evolution among fish taxa as a function of phylogenetic separation (a). Reports were also categorised by the type of repeated evolution (b) and phenotypic characteristic studied (c). See Fig. 2 for other details. [file 12862_2015_424_MOESM2_ESM.pdf]

a)

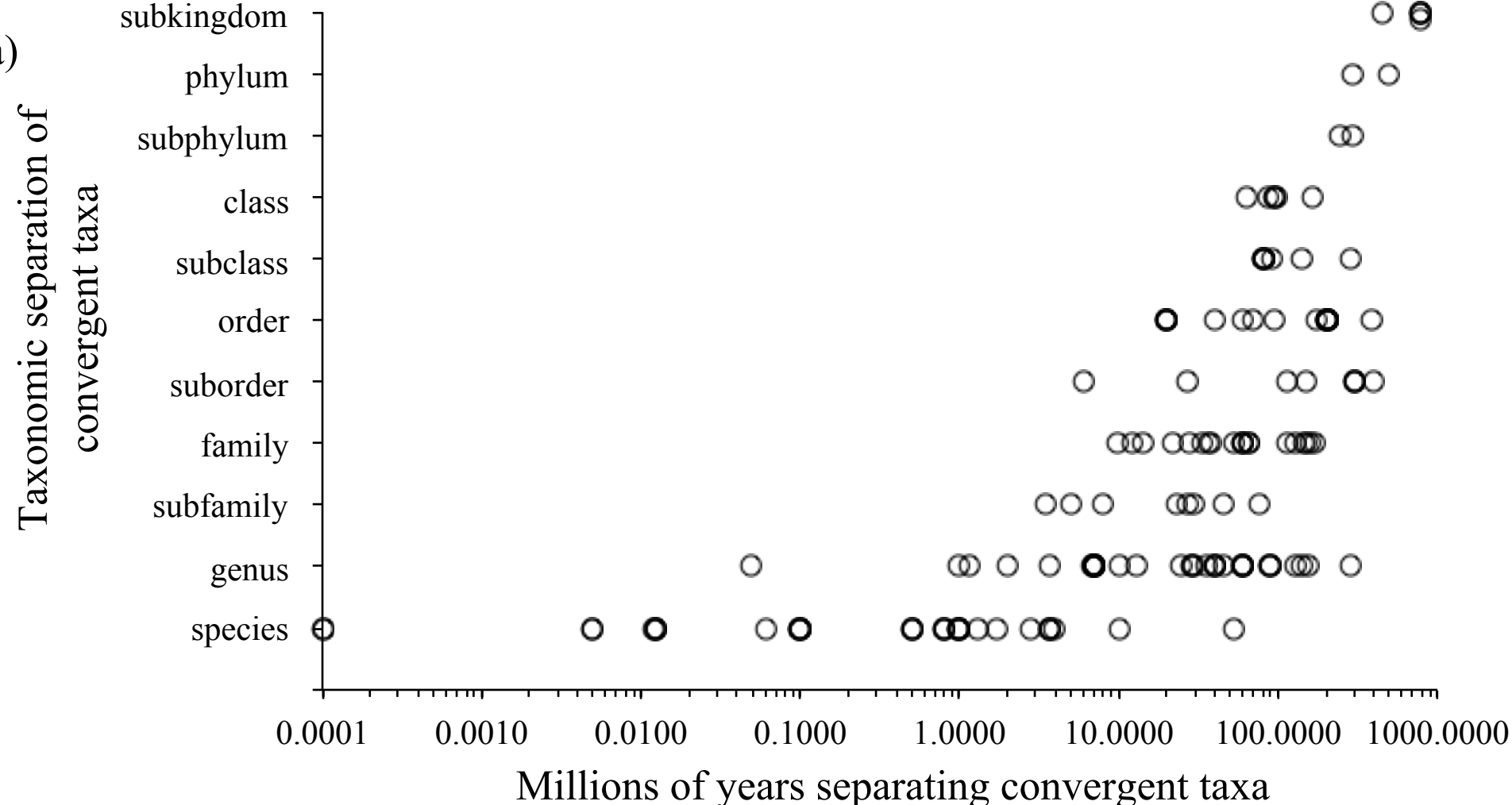

b)

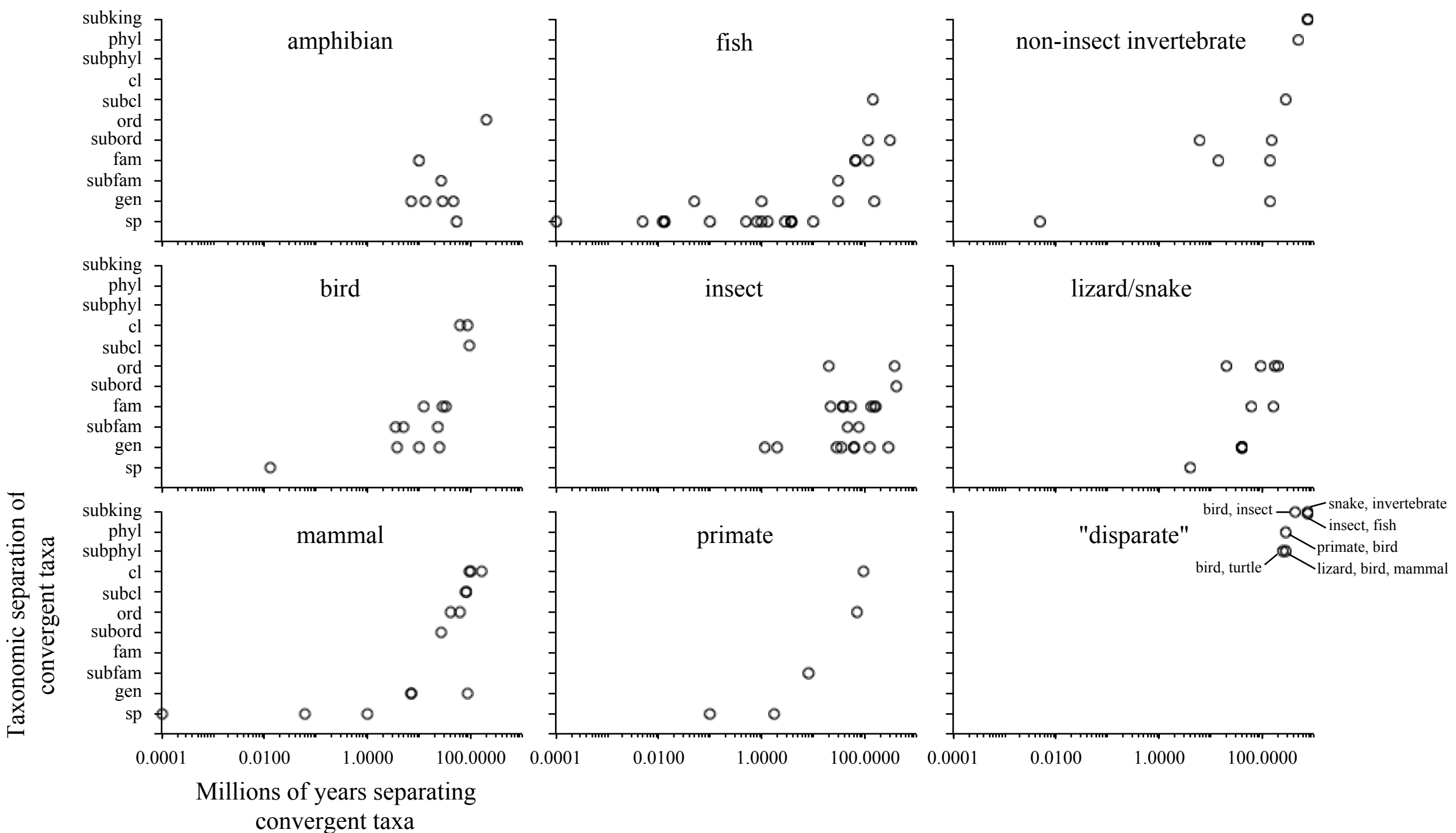

Supplement: Additional file 4: Figure S3. — The relationship between time and taxonomic level separating convergent taxa. Also shown are individual plots for major taxonomic groups included in the meta-analysis. [file 12862_2015_424_MOESM4_ESM.pdf]
