# Supplementary material for: Repeated evolution and the impact of evolutionary history on adaptation
Source: BMC Evol Biol. 2015 Jul 10;15:137. doi: 10.1186/s12862-015-0424-z (PMC4497378; doi:10.1186/s12862-015-0424-z)
Supplement: Additional file 3: Table S1. — Reported examples of adaptive convergence published over the last decade. [file 12862_2015_424_MOESM3_ESM.docx]

| **Table S1**  **Reported examples of adaptive convergence published over the last decade.** See also Conte et al. 2012 for additional parallel and convergent examples used in the meta-analysis. | | | | | | | | |
| --- | --- | --- | --- | --- | --- | --- | --- | --- |
| Report | Taxonomic distance | Millions of years separation | Type of organism | Phenotype | Selection pressure | Type of adaptation | Source | Notes |
| 1 | species/population | <20† | marine fish | life history |  | convergent | Waples et al. 2004 |  |
| 2 | species/population | <20† | freshwater fish | morphology, behavior, life history | habitat type/use | convergent | Palkovacs et al. 2008 |  |
| 3 | species/population | <20† | mollusc | morphology |  | convergent | Panova et al. 2006 |  |
| 4 | species/population | <20† | freshwater fish | morphology | habitat type/use | convergent | Deagle et al. 2012 |  |
| 5 | species/population | <20† | freshwater fish | morphology | habitat type/use | convergent | Landry and Bernatchez 2010 | see also 12 |
| 6 | species/population | <20† | freshwater fish | morphology, life history | habitat type/use | parallel | Macqueen et al. 2011 |  |
| 7 | species/population | <20† | bird | morphology, behavior | habitat type/use, sexual selection | convergent | Odeen and Bjorklund 2003 |  |
| 8 | species/population | <20† | freshwater fish | morphology | habitat type/use | convergent | Kano et al. 2012 |  |
| 9 | species/population | <20† | mammal | morphology | predation | functional redundancy | Hoekstra et al. 2006 |  |
| 10 | species/population | <20† | marine fish | physiology | habitat type/use | parallel | Bradbury et al. 2010 |  |
| 11 | species/population | <20† | mammal | morphology | predation | functional redundancy | Nachman et al. 2003 |  |
| 12 | species/population | <20† | freshwater fish | morphology | habitat type/use | convergent | Landry et al. 2007 | see also 5 |
| 13 | species/population | <20† | primate | morphology | habitat type/use | convergent | Norton et al. 2006 |  |

| **Table S1:** Continued. | | | | | | | | |
| --- | --- | --- | --- | --- | --- | --- | --- | --- |
| Report | Taxonomic distance | Millions of years separation | Type of organism | Phenotype | Selection pressure | Type of adaptation | Source | Notes |
| 14 | species/population | <20† | marine fish, freshwater fish | morphology | habitat type/use | convergent | Herczeg et al. 2010 |  |
| 15 | species/population | <20‡ | freshwater fish | morphology | habitat type/use | convergent | Laporte et al. 2011 |  |
| 16 | species/population | <20‡ | freshwater fish | morphology, behavior | predation, sexual selection | convergent | Hendry et al. 2006 | see also 17 |
| 17 | species/population | <20‡ | freshwater fish | behavior | predation, sexual selection | convergent | Schwartz and Hendry 2007 | see also 16 |
| 18 | species/population | <20‡ | freshwater fish | behavior | habitat type/use | convergent | Plath and Schlupp 2008 |  |
| 19 | species/population | <20‡ | lizard | life history | habitat type/use | convergent | Oufieroi and Angilletta 2006 |  |
| 20 | species/population | 40-60‡ | amphibian | morphology, behavior | habitat type/use | convergent | Rice et al. 2009 |  |
| 21 | species/population |  | mammal | physiology |  | functional redundancy | Guderley et al. 2006 |  |
| 22 | genus | <20† | freshwater fish | morphology | habitat type/use | convergent | Miyazaki et al. 2011 |  |
| 23 | genus | <20† | insect | morphology, behavior | predation, sexual selection | convergent | Stoks et al. 2005 |  |
| 24 | genus | <20† | insect | morphology | habitat type/use | convergent | de Busschere et al. 2012 |  |
| 25 | genus | <20† | bird | behavior | sexual selection | convergent | Laiolo 2012 |  |
| 26 | genus | <20† | amphibian | morphology | habitat type/use | convergent | Adams 2010 |  |
| 27 | genus | <20† | bird | morphology |  |  | Grant et al. 2004 |  |

| **Table S1:** Continued. | | | | | | | | |
| --- | --- | --- | --- | --- | --- | --- | --- | --- |
| Report | Taxonomic distance | Millions of years separation | Type of organism | Phenotype | Selection pressure | Type of adaptation | Source | Notes |
| 28 | genus | 20-40† | insect | morphology | habitat type/use | convergent | Chapman et al. 2006 |  |
| 29 | genus | 20-40† | insect | morphology |  | convergent | Wood et al. 2007 |  |
| 30 | genus | 20-40‡ | bird | morphology | habitat type/use | convergent | Jonsson et al. 2009 |  |
| 31 | genus | 20-40‡ | amphibian | morphology | predation | convergent | Chiari et al. 2004 |  |
| 32 | genus | 20-40‡ | freshwater fish | morphology | sexual selection | convergent | Duftner et al. 2007 |  |
| 33 | genus | 40-60† | lizard | morphology | habitat type/use | convergent | Kolbe et al. 2011 | see also 34, 35, and 72 |
| 34 | genus | 40-60‡ | lizard | morphology, behavior | habitat type/use | convergent | Ord et al. 2013 | see also 33, 35 and 72 |
| 35 | genus | 40-60‡ | lizard | morphology | habitat type/use | functional redundancy | Toro et al. 2004 | see also 33, 34 and 72 |
| 36 | genus | 60-80‡ | insect | morphology | sexual selection | convergent | Atallah et al. 2009 |  |
| 37 | genus | 120-140‡ | insect | morphology | food type | convergent | Larkin et al. 2006 |  |
| 38 | genus | 140-160‡ | crustacean | life history | habitat type/use | convergent | Murphy and Austin 2005 |  |
| 39 | genus | 140-160‡ | freshwater fish | physiology | habitat type/use | convergent | Wilkens and Strecker 2003 |  |
| 40 | genus | 280-300‡ | insect | morphology, behavior, life history | habitat type/use | parallel | Henry et al. 2012 |  |

| **Table S1:** Continued. | | | | | | | | |
| --- | --- | --- | --- | --- | --- | --- | --- | --- |
| Report | Taxonomic distance | Millions of years separation | Type of organism | Phenotype | Selection pressure | Type of adaptation | Source | Notes |
| 41 | genus |  | bird | morphology | habitat type/use | convergent | Prager and Andersson 2010 |  |
| 42 | genus |  | bird | morphology, behavior | sexual selection | convergent | Price et al. 2007 |  |
| 43 | subfamily/tribe | <20† | bird | morphology | habitat type/use | convergent | Haring et al. 2007 |  |
| 44 | subfamily/tribe | <20† | bird | morphology | sexual selection | convergent | Moore et al. 2006 |  |
| 45 | subfamily/tribe | <20‡ | primate | morphology |  | convergent | Leigh 2007 |  |
| 46 | subfamily/tribe | 20-40‡ | bird | morphology, behavior |  | convergent | Aliabadian et al. 2012 |  |
| 47 | subfamily/tribe | 20-40‡ | amphibian | morphology | predation | convergent | Vences et al. 2003 |  |
| 48 | subfamily/tribe | 20-40‡ | freshwater fish | physiology | habitat type/use | parallel | O'Quin et al. 2010 |  |
| 49 | subfamily/tribe | 40-60† | insect | morphology, physiology | habitat type/use | convergent | Kergoat et al. 2005 |  |
| 50 | subfamily/tribe | 60-80‡ | insect | physiology | predation | convergent | Bunnige et al. 2008 |  |
| 51 | family | <20† | bird | morphology |  | convergent | Holland et al. 2010 |  |
| 52 | family | <20† | mollusc | behavior, physiology | habitat type/use | convergent | Haase 2005 |  |
| 53 | family | <20‡ | amphibian | morphology, behavior | habitat type/use | convergent | Kohler and Gunther 2008 |  |
| 54 | family | 20-40† | insect | morphology | habitat type/use | convergent | Buckley et. al. 2009 |  |
| 55 | family | 20-40† | bird | morphology, behavior | habitat type/use | convergent | Irestedt et al. 2009 |  |

| **Table S1:** Continued. | | | | | | | | |
| --- | --- | --- | --- | --- | --- | --- | --- | --- |
| Report | Taxonomic distance | Millions of years separation | Type of organism | Phenotype | Selection pressure | Type of adaptation | Source | Notes |
| 56 | family | 20-40† | insect | physiology | food type | convergent | Su et al. 2007 |  |
| 57 | family | 20-40† | insect | morphology | habitat type/use | convergent | Hedin and Thomas 2010 |  |
| 58 | family | 20-40‡ | bird | physiology | habitat type/use | functional redundancy | McCracken et al. 2009 |  |
| 59 | family | 40-60† | insect | morphology, behavior | predation | convergent | Ge et al. 2011 |  |
| 60 | family | 60-80‡ | marine fish | morphology | food type | functional redundancy | Hulsey and Wainwright 2002 | see also 61 |
| 61 | family | 60-80‡ | marine fish | morphology | food type | functional redundancy | Wainwright et al. 2003 | see also 60 |
| 62 | family | 100-120‡ | marine fish | morphology | food type | functional redundancy | Alfaro et al. 2004 |  |
| 63 | family | 140-160† | mollusc | morphology, life history |  | convergent | Alejandrino et al. 2011 |  |
| 64 | family | 140-160‡ | insect | morphology | predation | convergent | Oxford 2009 |  |
| 65 | family | 140-160‡ | lizard | physiology |  | functional redundancy | Stewart and Thompson 2009 |  |
| 66 | family |  | freshwater fish | behavior | habitat type/use | functional redundancy | Blob et al. 2006 |  |
| 67 | suborder/superfamily | <20† | crustacean | morphology | habitat type/use | convergent | Daniels et al. 2006 |  |
| 68 | suborder/superfamily | 20-40‡ | mammal | physiology | habitat type/use | convergent | Xu et al. 2008 |  |
| 69 | suborder/superfamily | 100-120‡ | marine fish, freshwater fish | morphology | food type | functional redundancy | Wainwright and Richard 1995 |  |
| 70 | suborder/superfamily | 140-160‡ | crustacean | morphology | habitat type/use | convergent | Fratini et al. 2005 |  |

| **Table S1** Continued. | | | | | | | | |
| --- | --- | --- | --- | --- | --- | --- | --- | --- |
| Report | Taxonomic distance | Millions of years separation | Type of organism | Phenotype | Selection pressure | Type of adaptation | Source | Notes |
| 71 | suborder/superfamily | 400-420† | insect | morphology, physiology | predation | functional redundancy | Pachl et al. 2012 | see also 78 |
| 72 | order | 20-40† | lizard | morphology | habitat type/use | functional redundancy | Warheit et al. 1999 | see also 33, 34 and 35 |
| 73 | order | 40-60† | mammal | morphology | food type | functional redundancy | Tseng and Wang 2011 |  |
| 74 | order | 60-80‡ | mammal | physiology | food type | parallel | Liu et al. 2012 |  |
| 75 | order | 60-80‡ | primate | physiology | food type | convergent | Pampush et al. 2013 |  |
| 76 | order | 80-100‡ | lizard | morphology | food type | functional redundancy | Stayton 2006 |  |
| 77 | order | 160-180† | lizard | morphology | predation | convergent | Rosenblum 2006 |  |
| 78 | order | 380-400† | insect | morphology | habitat type/use | convergent | Maraun et al. 2009 | see also 71 |
| 79 | subclass/superorder | 80-100‡ | mammal | physiology | food type; sexual selection | parallel | Davies et al. 2012 |  |
| 80 | subclass/superorder | 80-100‡ | bird | morphology | food type | convergent | Jones et al. 2012 |  |
| 81 | subclass/superorder | 140-160† | marine fish | morphology | habitat type/use | convergent | Aschliman et al. 2012 |  |
| 82 | subclass/superorder | 280-300‡ | mollusc | physiology | habitat type/use | convergent | Lindgren et al. 2012 |  |
| 83 | class | 80-100‡ | bird | morphology, physiology | food type | convergent | Fidler et al. 2004 |  |
| 84 | class | 80-100‡ | mammal | physiology |  | convergent | Wicher and Fries 2007 |  |
| 85 | class | 80-100‡ | primate | morphology |  | functional redundancy | Raichlen 2004 |  |
| 86 | class | 80-100‡ | mammal | physiology |  | convergent | Emera et al. 2012 |  |

| **Table S1:** Continued. | | | | | | | | |
| --- | --- | --- | --- | --- | --- | --- | --- | --- |
| Report | Taxonomic distance | Millions of years separation | Type of organism | Phenotype | Selection pressure | Type of adaptation | Source | Notes |
| 87 | class | 160-180‡ | mammal | morphology | food type | convergent | Wroe and Milne 2007 |  |
| 88 | superclass | 240-260‡ | turtle, bird | physiology | food type | functional redundancy | Tokita et al. 2012 |  |
| 89 | superclass | 280-300‡ | lizard, bird, mammal | behavior, life history |  | convergent | Davis et al. 2011 |  |
| 90 | subphylum/phylum | 280-300‡ | primate, bird | physiology | habitat type/use | functional redundancy | Menegaz and Kirk 2009 |  |
| 91 | subphylum/phylum | 480-500† | mollusc | physiology | predation | functional redundancy | Jackson et al. 2009 |  |
| 92 | subkingdom/kingdom | 440-460‡ | bird, insect | behavior, life history | host type | convergent | Cervo et al. 2005 |  |
| 93 | subkingdom/kingdom | 780-800‡ | insect, freshwater fish | physiology | habitat type/use | convergent | Bilandzija et al. 2012 |  |
| 94 | subkingdom/kingdom | 780-800‡ | mollusc | physiology |  | convergent | Yoshida et al. 2010 |  |
| 95 | subkingdom/kingdom | 780-800‡ | snake, crustacean | morphology, behavior | food type | convergent | Hoso et al. 2007 |  |
| 96 | subkingdom/kingdom | 780-800‡ | marine invertebrate | behavior | habitat type/use | functional redundancy | Koehl 1996 |  |

†from source

‡from TimeTree

**Literature Cited in Table S1**

Adams, D. C. 2010. Parallel evolution of character displacement driven by competitive selection in terrestrial salamanders. BMC Evol. Biol. 10: 72.

Alejandrino, A., L. Puslednik & J. M. Serb. 2011. Convergent and parallel evolution in life habit of the scallops (Bivalvia: Pectinidae). BMC Evol. Biol. 11: 164.

Alfaro, M. E., D. I. Bolnick & P. C. Wainwright. 2004. Evolutionary dynamics of complex biomechanical systems: an example using the four-bar mechanism. Evolution 58: 495-503.

Aliabadian, M., M. Kaboli, M. I. Forschler, V. Nijman, A. Chamani, A. Tillier, R. Prodon, E. Pasquet, G. P. Ericson & D. Zuccon. 2012. Convergent evolution of morphological and ecological traits in the open-habitat chat complex (Aves, Muscicapidae: Saxicolinae). Mol. Phylogenet. Evol. 65: 35-45.

Aschliman, N. C., M. Nishida, M. Miya, J. G. Inoue, K. M. Rosana & G. J. Naylor. 2012. Body plan convergence in the evolution of skates and rays (Chondrichthyes: Batoidea). Mol. Phylogenet. Evol. 63: 28-42.

Atallah, J., N. H. Liu, P. Dennis, A. Hon & E. W. Larsen. 2009. Developmental constraints and convergent evolution in *Drosophila* sex comb formation. Evol. Dev. 11: 205-218.

Bilandzija, H., H. Cetkovic & W. R. Jeffery. 2012. Evolution of albinism in cave planthoppers by a convergent defect in the first step of melanin biosynthesis. Evol. Devo. 14: 196-203.

Blob, R. W., R. Rai, M. L. Julius & H. L. Schoenfuss. 2006. Functional diversity in extreme environments: effects of locomotor style and substrate texture on the waterfall-climbing performance of Hawaiian gobiid fishes. J. Zool. 268: 315-324.

Bradbury, I. R., S. Hubert, B. Higgins, T. Borza, S. Bowman, I. G. Paterson, P. V. Snelgrove, C. J. Morris, R. S. Gregory, D. C. Hardie, J. A. Hutchings, D. E. Ruzzante, C. T. Taggart & P. Bentzen. 2010. Parallel adaptive evolution of Atlantic cod on both sides of the Atlantic Ocean in response to temperature. Proc. Roy. Soc. Biol. Sci. 277: 3725-3734.

Buckley, T. R., D. Attanayake & S. Bradler. 2009. Extreme convergence in stick insect evolution: phylogenetic placement of the Lord Howe Island tree lobster. Proc. Roy. Soc. Biol. Sci. 276: 1055-1062.

Bunnige, M., M. Hilker & S. Dobler. 2008. Convergent evolution of chemical defence in Galerucine larvae. Biol. J. Linn. Soc. 93: 165-175.

Cervo, R., V. Macinai, F. Dechigi & S. Turillazzi. 2005. Fast growth of immature brood in a social parasite wasp: a convergent evolution between avian and insect cuckoos. Am. Nat. 164: 814-820.

Chapman, E. G., B. A. Foote, J. Malukiewicz & W. R. Hoeh. 2006. Parallel evolution of larval morphology and habitat in the snail-killing genus *Tetanocera*. J. Evol. Biol. 19: 1459-1474.

Chiari, Y., M. Vences, D. R. Vieites, F. Rabemananjara, P. Bora, O. Ramilijaona Ravoahangimalala & A. Meyer. 2004. New evidence for parallel evolution of colour patterns in Malagasy poison frogs (Mantella). Mol. Ecol. 13: 3763-3774.

Conte, G. L., M. E. Arnegard, C. L. Peichel & D. Schluter. 2012. The probability of genetic parallelism and convergence in natural populations. Proc. Roy. Soc. Lond. B 279: 5039-5047.

Daniels, S. R., N. Cumberlidge, M. Perez-Losada, S. A. Marijnissen & K. A. Crandall. 2006. Evolution of Afrotropical freshwater crab lineages obscured by morphological convergence. Mol. Phylogenet. Evol. 40: 227-235.

Davies, K. T., J. A. Cotton, J. D. Kirwan, E. C. Teeling & S. J. Rossiter. 2012. Parallel signatures of sequence evolution among hearing genes in echolocating mammals: an emerging model of genetic convergence. Heredity 108: 480-489.

Davis, A. R., A. Corl, Y. Surget-Groba & B. Sinervo. 2011. Convergent evolution of kin-based sociality in a lizard. Proc. Roy. Soc. Biol. Sci. 278: 1507-1514.

de Busschere C., L. Baert, S. M. van Belleghem, W. Dekoninck & F. Hendrickx. 2012. Parallel phenotypic evolution in a wolf spider radiation on Galapagos. Biol. J. Linn. Soc. 106: 123-136.

Deagle B. E., F. C. Jones, Y. F. Chan, D. M. Absher, D. M. Kingsley & T. E. Reimchen. 2012. Population genomics of parallel phenotypic evolution in stickleback across stream-lake ecological transitions. Proc. Roy. Soc. Biol. Sci. 279: 1277-1286.

Duftner, N., K. M. Sefc, S. Koblmuller, W. Slazburger, M. Taborsky & C. Sturmbauer. 2007. Parallel evolution of facial stripe patterns in the *Neolamprologus brichardi/pulcher* species complex endemic to Lake Tanganyika. Mol. Phylogenet. Evol. 45: 706-715.

Emera, D., C. Casola, V. J. Lynch, D. E. Wildman, D. Agnew & G. P. Wagner. 2012. Convergent evolution of endometrial prolactin expression in primates, mice, and elephants through the independent recruitment of transposable elements. Mol. Biol. Evol. 29: 239-247.

Fidler, A. E., S. Kuhn & E. Gwinner. 2004. Convergent evolution of strigiform and caprimulgiform dark-activity is supported by phylogenetic analysis using the arylalkylamine N-acetyltransferase (Aanat) gene. Mol. Phylogenet. Evol. 33: 908-921.

Fratini, S., M. Vannini, S. Cannicci & C. D. Schubart. 2005. Tree-climbing mangrove crabs: a case of convergent evolution. Evol. Ecol. Res. 7: 219-233.

Ge, D., D. Chesters, J. Gómez-Zurita, L. Zhang, X. Yang & A. P. Vogler. 2011. Anti-predator defence drives parallel morphological evolution in flea beetles. Proc. Roy. Soc. Biol. Sci. 278: 2133-2141.

Grant, P. R., B. R. Grant, J. A. Markert, L. F. Keller & K Petren. 2004. Convergent evolution of Darwin's finches caused by introgressive hybridization and selection. Evolution 58: 1588-1599.

Guderley H., P. Houle-Leroy, G. M. Diffee, D. M. Camp, & T. Garland Jr. 2006. Morphometry, ultrastructure, myosin isoforms, and metabolic capacities of the “mini muscles” favoured by selection for high activity in house mice. Comp. Biochem. Physiol. B 144: 271-282.

Haase, M. 2005. Rapid and convergent evolution of parental care in hydrobiid gastropods from New Zealand. J. Evol. Biol. 18: 1076-1086.

Haring, E., K. Kvaloy, J. -O. Gjershaug, N. Rov & A. Gamauf. 2007. Convergent evolution and paraphyly of the hawk-eagles of the genus *Spizaetus* (Aves, Accipitridae) - phylogenetic analyses based on mitochondrial markers. J. Zool. Syst. Evol. Res. 45: 353-365.

Hedin, M. & S. M. Thomas. 2010. Molecular systematics of eastern North American Phalangodidae (Arachnida: Opiliones: Laniatores), demonstrating convergent morphological evolution in caves. Mol. Phylogenet. Evol. 54: 107-121.

Hendry, A. P., M. L. Kelly, M. T. Kinnison & D. N. Reznick. 2006. Parallel evolution of the sexes? Effects of predation and habitat features on the size and shape of wild guppies. J. Evol. Biol. 19: 741-754.

Henry, C. S., S. J. Brooks, P. Duelli, J. B. Johnson, M. M. Wells & A. Mochizuki. 2012. Parallel evolution in courtship songs of North American and European green lacewings (Neuroptera: Chrysopidae). Biol. J. Linn. Soc. 105: 776-796.

Herczeg, G., M. Turtiainen & J. Merila. 2010. Morphological divergence of North-European nine-spined sticklebacks (*Pungitius pungitius*): signatures of parallel evolution. Biol. J. Linn. Soc. 10: 403-416.

Hoekstra, H., E., R. J. Hirschmann, R. A. Bundey, P. A. Insel, & J. P. Crossland. 2006. A single amino acid mutation contributes to adaptive beach mouse color pattern. Science 313: 101-104.

Holland, B. R., H. G. Spencer, T. H. Worthy & M. Kennedy. 2010. Identifying cliques of convergent characters: concerted evolution in the cormorants and shags. Syst. Biol. 59: 433-445.

Hoso M., T. Asami & M. Hori. 2007. Right-handed snakes: convergent evolution of asymmetry for functional specialization. Biol. Lett. 3: 169-172.

Hulsey C. D. & P. C. Wainwright. 2002. Projecting mechanics into morphospace: disparity in the feeding system of labrid fishes. Proc. Roy. Soc. Biol. Sci. 269: 317-326

Irestedt, M., J. Fjeldsa, L. Dalen & P. G. Ericson. 2009. Convergent evolution, habitat shifts and variable diversification rates in the ovenbird-woodcreeper family (Furnariidae). BMC Evol. Biol. 9: 268.

Jackson, D. J. C. McDougall, B. Woodcroft, P. Moase, R. A. Rose, M. Kube, R. Reinhardt, D. S. Rokhsar, C. Montagnani, C. Joubert, D. Piquemal & B. M. Degnan. 2009. Parallel evolution of nacre building gene sets in molluscs. Mol. Biol. Evol. 27: 591-608.

Jones, L. R., H. L. Black & C. M. White. 2012. Evidence for the convergent evolution in gape morphology in the bat hawk (*Macheiramphus alcinus*) with swifts, swallows and goatsuckers. Biotropica 44: 386-393.

Jonsson, K. A., M. Irestedt, P. G. P. Ericson & J. Fjeldsa. 2009. A Molecular phylogeny of minivets (Passeriformes: Campephagidae: *Pericrocotus*): implications for biogeography and convergent plumage evolution. Zool. Scripta 39: 1-8.

Kano, Y., S. Nishida & J. Nakajima. 2012. Waterfalls drive parallel evolution in a freshwater goby. Ecol. Evol. 2: 1805-1817.

Kergoat, G. J., N. Alvarez, M. Hossaert-Mckey, N. Faure & F. Silvain. 2005. Parallels in the evolution of the two largest New and Old World seed-beetle genera (Coleoptera, Bruchidae). Mol. Ecol. 14: 4003-4021.

Koehl M. A. R. 1996. When does morphology matter? Ann. Rev. Ecol. Syst. 27: 501-542.

Kohler, F. & R. Gunther. 2008. The radiation of microhylid frogs (Amphilia: Anura) on New Guinea: A mitochondrial phylogeny revels parallel evolution of morphological and life history traits and disproves the current morphology-based classification. Mol. Phylogenet. Evol. 47: 353-365.

Kolbe, J. J., L. J. Revell, B. Szekely, E. D. Brodie 3^rd^ & J. B. Losos. 2011. Convergent evolution of phenotypic integration and its alignment with morphological diversification in Caribbean *Anolis* ecomorphs. Evolution 65: 3608-3624.

Laiolo, P. 2012. Interspecific interactions drive cultural co-evolution and acoustic convergence in syntopic species. J. Anim. Ecol. 81: 594-604.

Landry L. &L. Bernatchez. 2010. Role of epibenthic resource opportunities in the parallel evolution of lake whitefish species pairs (*Coregonus* sp.). J. Evol. Biol. 23: 2602-2613.

Landry, L., W. F. Vincent & L. Bernatchez. 2007. Parallel evolution of lake whitefish dwarf ecotypes in association with limnological features of their adaptive landscape. J. Evol. Biol. 20: 971-984.

Laporte, M., P. Magnan & B. Angers. 2011. Genetic differentiation between the blue and the yellow phenotypes of walleye (*Sander vitreus*): an example of parallel evolution. Ecoscience 18: 124-129.

Larkin L. L., J. L. Neff & B. B. Simpson. 2006. Phylogeny of the *Callandrena* subgenus of the Andrena (Hymenoptera: Andrenidae) based on mitochondrial and nuclear DNA data: polyphyly and convergent evolution. Mol. Phylogenet. Evol. 38: 330-343.

Leigh, S. R. 2007. Homoplasy and the evolution of ontogeny in papionin primates. J. Hum. Evol. 52: 536-558.

Lindgren, A. R., M. S. Pankey, F. G. Hochberg & T. H. Oakley. 2012. A multi-gene phylogeny of Cephalopoda supports convergent morphological evolution in association with multiple habitat shifts in the marine environment. BMC Evol. Biol. 12: 129.

Liu, Y., N. Han, L. F. Franchini, H. Xu, F. Pisciottano, A. B. Elgoyhen, K. E. Rajan & S. Zhang. 2012. The voltage-gated potassium channel subfamily KQT member 4 (KCNQ4) displays parallel evolution in echolocating bats. Mol. Biol. Evol. 29: 1441-1450.

Macqueen, D. J., B. K. Kristjansson, C. G. Paxton, V. L. Vieira & I. A. Johnston. 2011. The parallel evolution of dwarfism in Arctic charr is accompanied by adaptive divergence in mTOR-pathway gene expression. Mol. Ecol. 20: 3167-3184.

McCracken, K. G., C. P. Barger, M. Bulgarella, K. P. Johnson, S. A. Sonsthagen, J. Trucco, T. H. Valqui, R. E. Wilson, K. Winker & M. D. Sorenson. 2009. Parallel evolution in the major haemoglobin genes of eight species of Andean waterfowl. Mol. Ecol. 18: 3992-4005.

Maraun, M., G. Erdmann, G. Schulz, R. A. Norton, S. Scheu & K. Domes. 2009. Multiple convergent evolution of arboreal life in oribatid mites indicates the primacy of ecology. Proc. Roy. Soc. Biol. Sci*.* 276: 3219-3227.

Menegaz, R. A. & E. C. Kirk. 2009. Septa and processes: convergent evolution of the orbit in the haplorhine primates and strigiform birds. J. Hum. Evol. 57: 672-687.

Miyazaki, J., M. Dobashi, T. Tamura, S. Beppu, T. Sakai, M. Mihara & K Hosoya. 2011. Parallel evolution in eight-barbel loaches of the genus *Lefua* (Balitoridae, Cypriniformes) revealed by mitochondrial and nuclear DNA phylogenies. Mol. Phylogenet. Evol. 60: 416-427.

Moore, W. S., A. C. Weibel & A. Agius. 2006. Mitochondrial DNA phylogeny of the woodpecker genus *Veniliornis* (Picidae, Picinae) and related genera implies convergent evolution of plumage patterns. Biol. J. Linn. Soc. 87: 611-624.

Murphy, N. P. & C. M. Austin. 2005. Phylogenetic relationships of the globally distributed freshwater prawn genus *Macrobrachium* (Crustacea: Decapoda: Palaemonidae): biogeography, taxonomy and the convergent evolution of abbreviated larval development. Zool. Scripta 34: 187-197.

Nachman, M. W., H. E. Hoekstra & S. L. D’Agostino. 2003. The genetic basis of adaptive melanism in pocket mice. Proc. Natl. Acad. Sci. USA 100: 5268-5273.

Norton, H. L., R. A. Kittles, E. Parra, P. McKeigue, X. Mao, K. Cheng, V. A. Canfield, D. G. Bradley, B. McEvoy & M. D. Shriver. 2006. Genetic evidence for the convergent evolution of light skin in Europeans and East Asians. Mol. Biol. Evol. 24: 710-722.

O’Quin, K. E., C. M. Hofmann, H. A. Hofmann & K. C. Carleton. 2010. Parallel evolution of opsin gene expression in African cichlid fishes. Mol. Biol. Evol. 27: 2839-2854.

Odeen, A. & M. Bjorklund. 2003. Dynamics in the evolution of sexual traits: losses and gains, radiation and convergence in yellow wagtails (*Motacilla flava*). Mol. Ecol. 12: 2113-2130.

Ord, T. J., J. A. Stamps & J. B. Losos. 2013. Convergent evolution in the territorial communication of a classic adaptive radiation: Caribbean *Anolis* lizards. Anim. Behav. 85: 1415-1426.

Oufieroi, C. E. & M. J. Angilletta Jr. 2006. Convergent evolution of embryonic growth and development in the eastern fence lizard (*Sceloporus undulatus*). Evolution 60: 1066-1075.

Oxford, G. S. 2009. An exuberant, undescribed colour polymorphism in *Theridion californicum* (Araneae, Theridiidae): implications for a theridiid pattern ground plan and the convergent evolution of visible morphs. Biol. J. Linn. Soc. 96: 23-34.

Pachl, P., K. Domes, G. Shulz, R. A. Norton, S. Scheu, I. Schaefer & M. Maraun. 2012. Convergent evolution of defence mechanisms in oribatid mites (Acari, Oribatida) shows no "ghosts of predation past". Mol. Phylogenet. Evol. 65: 412-420.

Palkovacs, E. P., K. B. Dion, D. M. Post & A. Caccone. 2008. Independent evolutionary origins of landlocked alewife populations and rapid parallel evolution of phenotypic traits. Mol. Ecol. 17: 582-597.

Pampush J. D., A. C. Duque, B. R. Burrows, D. J. Daegling, W. F. Kenney & W. S. McGraw. 2013. Homoplasy and think enamel in primates. J. Hum. Evol. 64: 216-224.

Panova, M., J. Hollander & K. Johannesson. 2006. Site-specific genetic divergence in parallel hybrid zones suggests nonallopatric evolution of reproductive barriers. Mol. Ecol. 15: 4021-4031.

Ericson P. G. P. 2012. Evolution of terrestrial birds in three continents: biogeography and parallel radiations. J. Biogeog. 39: 813-824.

Plath, M. & I. Schlupp. 2008. Parallel evolution leads to reduced shoaling behaviour in two cave dwelling populations of Atlantic mollies (*Poecilia mexicana*, Poeciliidae, Teleostei). Environ. Biol. Fish. 82: 289-297.

Prager, M. & S. Andersson. 2010. Convergent evol0ution of red carotenoid coloration in widowbirds and bishops (*Euplectes* spp.). Evolution 64: 3609-3619.

Price, J. J., N. R. Friedman, & K. E. Omland. 2007. Song and plumage evolution in the new world Orioles (*Icterus*) show similar lability and convergence in patterns. Evolution 61: 850-863.

Raichlen, D. A. 2004. Convergence of forelimb and hindlimb Natural Pendular Period in baboons (*Papio cynocephalus*) and its implication for the evolution of primate quadrupedalism. J. Hum. Evol. 46: 719-738.

Rice, A. M., A. R. Leichty & D. W. Pfenning. 2009. Parallel evolution and ecological selection: replicated character displacement in spadefoot toads. Proc. Roy. Soc. Biol. Sci. 276: 4189-4196.

Rosenblum, E. B. 2006. Convergent evolution and the divergent selection: Lizards at the White Sands ecotone. Am. Nat. 167: 1-15.

Schwartz, A. K. & A. P. Hendry. 2007. A test for the parallel co-evolution of male colour and female preference in Trinidadian guppies (*Poecilia reticulata*). Evol. Ecol. Res. 9: 71-90.

Stayton, C. T. 2006. Testing hypothesis of convergence with multivariate data: morphological and functional convergence among herbivorous lizards. Evolution 60: 824-841.

Stewart J. R. & M. B. Thompson. 2009. Parallel evolution in placentation in Australian scincid lizards. J. Exp. Zool. B. Mol. Dev. Evol. 312: 590-602.

Stoks R., J. L. Nystrom, M. L. May, & M. A. McPeek. 2005. Parallel evolution in ecological and reproductive traits to produce cryptic damselfly species across the Holarctic. Evolution 59: 1976-1988.

Su, K. F., R. Meier, R. R. Jackson, D. P. Harland & D. Li. 2007. Convergent evolution of eye ultrastructure and divergent evolution of vision-mediated predatory behaviour in jumping spiders. J. Evol. Biol. 20: 1478-1489.

Tokita M., W. Chaeychomsri & J. Siruntawineti. 2012. Developmental basis of toothlessness in turtles: Insight into convergent evolution of vertebrate morphology. Evolution 67: 260-273.

Toro, E., A. Herrel & D. Irschick. 2004. The evolution of jumping performance in Caribbean *Anolis* lizards: solutions to biomechanical trade-offs. Am. Nat. 163: 844-856.

Tseng, Z. J. & X. Wang. 2011. Do convergent ecomorphs evolve through convergent morphological pathways? Cranial shape evolution in fossil hyaenids and borophagine canids (Carnivora, Mammalia). Paleobiology 37: 470-489.

Vences, M., J. Kosuch, R. Boistel, C. F. B. Haddad, E. L. Marca, S. Lotters & M. Veith. 2003. Convergent evolution of aposematic coloration in Neotropical poison frogs: a molecular phylogenetic perspective. Organs. Divers. Evol. 3: 215-226.

Wainwright, P. C., D. R. Bellwood, M. W. Westneat, J. R. Grubich & A. S. Hoey. 2003. A functional morphospace for the skull of labrid fishes: patterns of diversity in a complex biomechanical system. Biol. J. Linn. Soc. 82: 1-25.

Wainwright, P. C. & B. A. Richard. 1995. Predicting patterns of prey use from morphology of fishes. Environ. Biol. Fish. 44: 97-113.

Waples, R. S., D. J. Teel, J. M. Myers & A. R. Marshall. 2004. Life-history divergence in Chinook salmon: historic contingency and parallel evolution. Evolution 58: 386-403.

Warheit, K. I., J. D. Forman, J. B. Losos & D. B. Miles. 1999. Morphological diversification and adaptive radiation: A comparison of two diverse lizard clades. Evolution 53: 1226-1234.

Wicher, K. B. & E. Fries. 2007. Convergent evolution of human and bovine haptoglobin: partial duplication of the genes. J. Mol. Evol. 65: 373-379.

Wilkens, H. & U. Strecker. 2003. Convergent evolution of the cavefish *Astyanax* (Characidae, Teleostei): genetic evidence from reduced eye-size and pigmentation. Biol. J. Linn. Soc. 80: 545-554.

Wood, H. M., C. E. Griswold & G. S. Spicer. 2007. Phylogenetic relationships wtihin an endemic group of Malagasy 'assassin spiders' (Araneae, Archaeidae): ancestral character reconstruction, convergent evolution and biogeography. Mol. Phylogenet. Evol. 45: 612-619.

Wroe S. & N. Milne. 2007. Convergence and remarkably consistent constraint in the evolution of carnivore skull shape. Evolution 61: 1251-1260.

Xu, S., B. Chen, K. Zhou & G. Yang. 2008. High similarity at three MHC loci between the baiji and finless porpoise: trans-species or convergent evolution? Mol. Phylogenet. Evol. 47: 36-44.

Yoshida, M. A., S. Shigeno, K. Tsuneki & H. Furuya. 2010. Squid vascular endothelial growth factor receptor: a shared molecular signature in the convergent evolution of closed circulatory systems. Evol. Dev. 12: 25-33.
