# Supplementary material for: Repeated evolution and the impact of evolutionary history on adaptation
Source: BMC Evol Biol. 2015 Jul 10;15:137. doi: 10.1186/s12862-015-0424-z (PMC4497378; doi:10.1186/s12862-015-0424-z)
Supplement: Additional file 5: Table S2. — Full model outputs from analyses presented in figures. [file 12862_2015_424_MOESM5_ESM.docx]

**Table S2 Model outputs for analyses presented in Figures 2, S1 and S2.**

| **Type of repeated evolution/characteristic** | **Estimate** | **Lower 95% CI, upper 95% CI** | ***z*** | ***P* value** |
| --- | --- | --- | --- | --- |
| **A. Time since separation, all taxa** | | |  |  |
| any (*N*_reports_ = 103) | |  |  |  |
| intercept | 2.80 | 2.49, 3.10 | 18.14 | < 0.0001 |
| exponent | -0.0058 | -0.0080, - 0.0039 | -5.53 | < 0.0001 |
| parrallel (*N*_reports_ = 17) | |  |  |  |
| intercept | 1.50 | 0.77, 2.15 | 4.30 | < 0.0001 |
| exponent | -0.0048 | -0.0121, 0.0007 | -1.50 | 0.13 |
| convergent (*N*_reports_ *=* 68) | |  |  |  |
| intercept | 2.52 | 2.15, 2.88 | 13.59 | < 0.0001 |
| exponent | -0.0058 | -0.0086, -0.0034 | -4.42 | < 0.0001 |
| functionally redundant (*N*_reports_ = 18) | | |  |  |
| intercept | 1.01 | 0.33, 1.61 | 3.11 | < 0.01 |
| exponent | -0.0021 | -0.0052, 0.0003 | -1.52 | 0.13 |
| morphology (*N*_reports_ = 68) | |  |  |  |
| intercept | 3.02 | 2.61, 3.40 | 15.02 | < 0.0001 |
| exponent | -0.0123 | -0.0166, -0.0084 | -5.86 | < 0.0001 |
| behavior (*N*_reports_ = 19) | |  |  |  |
| intercept | 1.56 | 0.95, 2.09 | 5.41 | < 0.0001 |
| exponent | -0.0019 | -0.0043, -0.000005 | -1.76 | 0.08 |
| physiology (*N*_reports_ *=* 29) | |  |  |  |
| intercept | 1.31 | 0.77, 1.79 | 5.07 | < 0.0001 |
| exponent | -0.0017 | -0.0039, 0.0002 | -1.60 | 0.11 |

**Table S2 Continued.**

| **Type of repeated evolution/characteristic** | **Estimate** | **Lower 95% CI, upper 95% CI** | ***z*** | ***P* value** |
| --- | --- | --- | --- | --- |
| **B. Taxonomic separation, all taxa** | | |  |  |
| any (*N*_reports_ = 108) | |  |  |  |
| intercept | 3.39 | 3.04, 3.72 | 19.64 | < 0.0001 |
| exponent | -0.23 | -0.31, -0.17 | -6.50 | < 0.0001 |
| parrallel (*N*_reports_ = 18) | |  |  |  |
| intercept | 1.63 | 0.69, 2.45 | 3.68 | < 0.001 |
| exponent | -0.15 | -0.38, 0.07 | -1.29 | 0.20 |
| convergent (*N*_reports_ *=* 70) | |  |  |  |
| intercept | 3.02 | 2.59, 3.42 | 14.3 | < 0.0001 |
| exponent | -0.23 | -0.33, -0.14 | -4.98 | < 0.0001 |
| functionally redundant (*N*_reports_ = 20) | | |  |  |
| intercept | 1.30 | 0.40, 2.07 | 3.10 | < 0.01 |
| exponent | -0.09 | -0.23, 0.04 | -1.29 | 0.20 |
| morphology (*N*_reports_ = 70) | |  |  |  |
| intercept | 3.25 | 2.83, 3.65 | 15.46 | < 0.0001 |
| exponent | -0.29 | -0.40, -0.19 | -5.4 | < 0.0001 |
| behavior (*N*_reports_ = 21) | |  |  |  |
| intercept | 1.67 | 0.96, 2.31 | 4.88 | < 0.0001 |
| exponent | -0.10 | -0.24, 0.03 | -1.39 | 0.16 |
| physiology (*N*_reports_ *=* 30) | |  |  |  |
| intercept | 1.44 | 0.69, 2.10 | 4.02 | < 0.0001 |
| exponent | -0.08 | -0.20, 0.04 | -1.32 | 0.19 |

**Table S2 Continued.**

| **Type of repeated evolution/characteristic** | **Estimate** | **Lower 95% CI, upper 95% CI** | ***z*** | ***P* value** |
| --- | --- | --- | --- | --- |
| **C. Time since separation, fish only** | | |  |  |
| any (*N*_reports_ = 36) | |  |  |  |
| intercept | 2.49 | 1.89, 3.05 | 8.43 | < 0.0001 |
| exponent | -0.0138 | -0.0219, -0.0071 | -3.68 | < 0.001 |
| parrallel (*N*_reports_ = 14) | |  |  |  |
| intercept | 1.53 | 0.72, 2.27 | 3.93 | < 0.0001 |
| exponent | -0.0079 | -0.0187, -0.0007 | -1.80 | 0.07 |
| convergent (*N*_reports_ *=* 18) | |  |  |  |
| intercept | 2.37 | 1.64, 3.03 | 6.78 | < 0.0001 |
| exponent | -0.0147 | -0.0263, -0.0059 | -2.91 | < 0.01 |
| functionally redundant (*N*_reports_ = 4) | | |  |  |
| intercept | - | - | - | - |
| exponent | - | - | - | - |
| morphology (*N*_reports_ = 23) | |  |  |  |
| intercept | 2.5 | 1.78, 3.18 | 7.06 | < 0.0001 |
| exponent | -0.0188 | -0.0314, -0.0089 | -3.31 | < 0.001 |
| behavior (*N*_reports_ = 4) | |  |  |  |
| intercept | - | - | - | - |
| exponent | - | - | - | - |
| physiology (*N*_reports_ *=* 10) | |  |  |  |
| intercept | 0.53 | -0.56, 1.47 | 1.05 | 0.29 |
| exponent | -0.0029 | -0.0120, 0.0043 | -0.71 | 0.48 |
